# Comparing whole‐genome shotgun sequencing and DNA metabarcoding approaches for species identification and quantification of pollen species mixtures

**DOI:** 10.1002/ece3.8281

**Published:** 2021-11-04

**Authors:** Karen L. Bell, Robert A. Petit, Anya Cutler, Emily K. Dobbs, J. Michael Macpherson, Timothy D. Read, Kevin S. Burgess, Berry J. Brosi

**Affiliations:** ^1^ Department of Environmental Sciences Emory University Atlanta Georgia USA; ^2^ Division of Infectious Diseases Department of Medicine Emory University Atlanta Georgia USA; ^3^ Department of Biology Chapman University Orange California USA; ^4^ Department of Biology Columbus State University Columbus Georgia USA; ^5^ Present address: School of Biological Sciences University of Western Australia Perth Australia; ^6^ Present address: CSIRO Land & Water and CSIRO Health & Biosecurity Floreat WA Australia; ^7^ Present address: Department of Biology Northern Kentucky University Highland Heights Kentucky USA; ^8^ Present address: 23andMe Mountain View California USA; ^9^ Present address: Department of Biology University of Washington Seattle Washington USA

**Keywords:** DNA barcoding, DNA metabarcoding, environmental DNA, metagenomics, pollen, whole‐genome shotgun sequencing

## Abstract

Molecular identification of mixed‐species pollen samples has a range of applications in various fields of research. To date, such molecular identification has primarily been carried out via amplicon sequencing, but whole‐genome shotgun (WGS) sequencing of pollen DNA has potential advantages, including (1) more genetic information per sample and (2) the potential for better quantitative matching. In this study, we tested the performance of WGS sequencing methodology and publicly available reference sequences in identifying species and quantifying their relative abundance in pollen mock communities. Using mock communities previously analyzed with DNA metabarcoding, we sequenced approximately 200Mbp for each sample using Illumina HiSeq and MiSeq. Taxonomic identifications were based on the Kraken *k*‐mer identification method with reference libraries constructed from full‐genome and short read archive data from the NCBI database. We found WGS to be a reliable method for taxonomic identification of pollen with near 100% identification of species in mixtures but generating higher rates of false positives (reads not identified to the correct taxon at the required taxonomic level) relative to *rbcL* and ITS2 amplicon sequencing. For quantification of relative species abundance, WGS data provided a stronger correlation between pollen grain proportion and sequence read proportion, but diverged more from a 1:1 relationship, likely due to the higher rate of false positives. Currently, a limitation of WGS‐based pollen identification is the lack of representation of plant diversity in publicly available genome databases. As databases improve and costs drop, we expect that eventually genomics methods will become the methods of choice for species identification and quantification of mixed‐species pollen samples.

## Graphical abstract

Whole‐genome shotgun (WGS) sequencing has theoretical advantages over DNA metabarcoding including more genetic information per sample and the potential for better quantitative matching. We tested the potential of WGS sequencing to identify and quantify plant species in mixtures of pollen. We found WGS to be a reliable method for taxonomic identification of pollen and to provide a stronger correlation between pollen grain proportion and sequence read proportion compared to DNA metabarcoding.

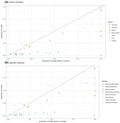

## 1 INTRODUCTION

The identification of species in pollen mixtures has a variety of applications, including forensics, paleobotany, allergen monitoring, and pollination biology. Identification of pollen by microscopy is limited as there are few experts on palynology (Rahl, 2008), many taxa cannot be identified to the species level based on pollen morphology (Khansari et al., 2012; Salmaki et al., 2008), and the methods are time‐consuming. Recent studies have used DNA metabarcoding to overcome these disadvantages. DNA metabarcoding uses high‐throughput sequencing methods to simultaneously sequence PCR‐amplified DNA of one or two molecular markers from all species in a mixture (Bell et al., 2016; Cristescu, 2014). DNA metabarcoding approaches have several advantages over microscopic identification, including higher taxonomic resolution in many instances, greater availability of relevant expertise, and the ability to process many more grains per sample and with higher throughput (Bell et al., 2019). Pollen DNA metabarcoding has been used for monitoring honey quality (Hawkins et al., 2015), determining the foraging patterns of bees (Keller et al., 2015; Richardson, Lin, Quijia, et al., 2015; Richardson, Lin, Sponsler, et al., 2015) and other pollinating insects (Lucas et al., 2018), monitoring allergenic pollen (Brennan et al., 2019; Kraaijeveld et al., 2014), and examining historical flower visitation (Gous et al., 2019).

Despite the successful use of pollen DNA metabarcoding, and its advantages relative to microscopic identification, metabarcoding has limitations. Species‐level identification may be impeded by a lack of divergence between related species in the DNA barcoding markers, while detection and quantification may be hampered by biases that favor certain species (Bell et al., 2019). Different species have different DNA isolation yields and vary in organellar or ribosomal genome copy number, which could lead to biases in DNA quantity going into PCRs (Kembel et al., 2012; Lamb et al., 2019; Pawluczyk et al., 2015). Primers for PCR may differ in their binding efficiencies to different species (Pompanon et al., 2012), or polymerases may be biased toward different nucleotide composition (Nichols et al., 2018), leading to PCR biases.

Whole‐genome shotgun (WGS) sequencing is an approach that could improve both taxonomic resolution and quantification in the molecular identification of pollen. In terms of taxonomic resolution, WGS sequences many more loci than DNA metabarcoding, even with very low coverage, generating the potential for much finer taxonomic resolution. In terms of quantification, WGS approaches do not require PCR and do not target particular gene regions, eliminating amplification bias and potentially reducing copy number bias (Bista et al., 2018). WGS methods are increasingly used to analyze the species composition and functional profiling of microbiomes (Sharpton, 2014; Venter et al., 2004) and, more recently, eukaryotes, including soil invertebrate communities (Andújar et al., 2015), herbivore diets (Chua et al., 2021; Srivathsan et al., 2014), organisms in honey (Bovo et al., 2018), and ancient plant communities (Parducci et al., 2019). The quantitative accuracy (e.g., Morgan et al., 2010) and species detection ability (Ranjan et al., 2016) of WGS has been investigated for prokaryote communities and more recently eukaryotic communities (Bista et al., 2018; Garrido‐Sanz et al., 2020; Gómez‐Rodríguez et al., 2015; Ji et al., 2019; Tang et al., 2014, 2015). However, few have examined the performance of WGS in identification and quantification of species in pollen mixtures, and most of these used only the small proportion of sequences from the plastid genome (Lang et al., 2019). Furthermore, there are two key limitations of WGS‐based pollen species identifications that have not been tested: Very few plants have had their whole genomes sequenced and publicly available genome sequence data may contain errors (Breitwieser et al., 2019).

In this study, we test the ability of WGS sequencing and current publicly available reference sequences to identify taxa and quantify their proportions in pollen mixtures. We specifically examined (1) the proportions of false‐negative identifications, that is, taxa present in the sample that were not identified; (2) the proportions of false‐positive identifications, that is, reads that were identified to taxa not present in the sample; and (3) quantitative matching, that is, the correspondence between the proportion of pollen grains in a sample and the proportion of sequence reads matching the respective plant species. We also examined the effect of sample complexity (in terms of species richness, relatedness of taxa, and rarity of pollen grains in a sample) on false‐negative and false‐positive identifications. Finally, we compared WGS to DNA metabarcoding in terms of performance in identification and quantification. Considering the current limitations of reference genome availability, completeness, and quality, we expected to find poorer taxonomic identification relative to metabarcoding. However, given the multiple sources of quantitative bias in amplicon sequencing, we expected to find improved quantification with WGS.

## 2 MATERIALS AND METHODS

### 2.1 Overview

Our overall approach—described in more detail in the subsequent paragraphs—was based on shotgun sequencing carefully constructed mixtures of pollen (a “mock community”). We had previously studied the efficacy of DNA metabarcoding with the same pollen mixtures (Bell et al., 2019), and to enhance interpretability, we conducted whole‐genome shotgun sequencing on *the exact same DNA isolations* that we had previously used for amplicon sequencing. We used the Kraken2 bioinformatics pipeline (Wood et al., 2019), which implements a *k*‐mer approach to taxonomically classify sequencing reads relative to a reference database, and subsequently analyzed the classified reads to assess the performance of WGS in terms of (1) false‐negative reads; (2) false‐positive reads; and (3) quantitative matching; both on its own and also relative to amplicon‐based methods.

### 2.2 Pollen mock communities

The mock communities of pollen we sequenced are described in full detail in Bell et al. (2019). We designed the samples to vary in (1) species richness (1–9 species); (2) relatedness of taxa within samples (from two species in the same genus, to species in widely disparate orders, and including all of the major angiosperm lineages); and (3) rarity of taxa within samples, ranging from approximately 5%–95% of pollen grains in a sample. The pollen mixtures were carefully quantified via microscopy, with several observers, to ensure that we were able to assess quantitative matching with high confidence. The nine species included in the mixtures cover a broad spectrum of the flowering plant phylogeny, including monocots and all subclasses of eudicots. We included 1–9 species in the mixtures as this represented the typical range of species richness in a pollen sample taken from an individual pollinator. Pollen mixtures were made from high‐purity pollen purchased from pharmaceutical companies, to minimize contamination with DNA from other organisms. Details of the suppliers and pollen mixtures are shown in Tables 1 and 2.

**TABLE 1:** Origin of species pollen samples used in this study

Species	Family	APG4 lineage	Haploid genome size	Supplier
*Populus tremuloides*	Salicaceae	Rosids	0.5 pg (Bai et al., 2012)	Sigma‐Aldrich Co
*Populus deltoides*	Salicaceae	Rosids	0.5 pg (Bai et al., 2012)	Sigma‐Aldrich Co
*Broussonetia papyrifera*	Moraceae	Rosids	0.7 pg (Ohri & Kumar, 1986)	Polysciences Inc
*Carya illinoinensis*	Juglandaceae	Rosids	0.83 pg (Bennett et al., 2000)	Polysciences Inc
*Bassia scoparia*	Amaranthaceae	Caryophyllales	1.12 pg (Kubesova et al., 2010)	Sigma‐Aldrich Co
*Ambrosia artemisiifolia*	Asteraceae	Asterids	1.16 (Kubesova et al., 2010)	Polysciences Inc
*Artemisia tridentata*	Asteraceae	Asterids	4.09 pg (Torrell & Valles, 2001)	Sigma‐Aldrich Co
*Poa pratensis*	Poaceae	Monocots	4.24 pg (Arumuganathan et al., 1999), 5.38 pg (Bennett et al., 1982)	Sigma‐Aldrich Co
*Zea mays*	Poaceae	Monocots	2.73 pg (Bennett & Smith, 1976)	Carolina Biological Supply

**TABLE 2:** Composition of artificial pollen mixtures used in this study

Mixture type	Mixture number	Species	Relative proportions in mixture (based on number of pollen grains)	Relative proportions in mixture (based on estimates of DNA quantity)	Sequencing facility and platform
Increasing species richness (2–9)	1	*Broussonetia papyrifera, Artemisia tridentata, Zea mays*	0.3235, 0.4504, 0.226	0.0843, 0.6859, 0.2298	EIGC HiSeq
	2	*Broussonetia papyrifera, Bassia scoparia, Artemisia tridentata, Poa pratensis, Zea mays*	0.4452, 0.0897, 0.2315, 0.1737, 0.06	0.1380, 0.0445, 0.4191, 0.3260, 0.0724	EIGC HiSeq
	3	*Populus tremuloides, Broussonetia papyrifera, Carya illinoinensis, Bassia scoparia, Artemisia tridentata, Poa pratensis, Zea mays*	0.1365, 0.2448, 0.1373, 0.0715, 0.1941, 0.1375, 0.078	0.0337, 0.0847, 0.0563, 0.0396, 0.3922, 0.2880, 0.1056	GGC MiSeq
	4	*Populus tremuloides, Populus deltoides, Broussonetia papyrifera, Carya illinoinensis, Bassia scoparia, Ambrosia artemisiifolia, Artemisia tridentata, Poa pratensis, Zea mays*	0.1782, 0.1782, 0.1571, 0.0695, 0.028, 0.13, 0.2327, 0.0802, 0.046	0.0458, 0.0458, 0.0565, 0.0296, 0.0161, 0.0775, 0.4892, 0.1748, 0.0647	GGC MiSeq
Decreasing taxonomic relatedness (congeneric‐different orders)	5	*Populus deltoides, Populus tremuloides*	Unknown		GGC MiSeq
	6	*Poa pratensis, Zea mays*	0.4919, 0.508	0.6006, 0.3994	GGC MiSeq
	7	*Broussonetia papyrifera, Artemisia tridentata*	0.411, 0.589	0.1067, 0.8933	GGC MiSeq
	8	*Broussonetia papyrifera, Poa pratensis*	0.5388, 0.4612	0.1617, 0.8383	GGC MiSeq
Increasing rarity of *Broussonetia papyrifera* (0.5388–0.0897 of pollen grains; 0.1617–0.0160 of DNA in pollen grains)	8	*Broussonetia papyrifera, Poa pratensis*	0.5388, 0.4612	0.1617, 0.8383	GGC MiSeq
	9	*Broussonetia papyrifera, Poa pratensis*	0.1514, 0.8486	0.0286, 0.9714	GGC MiSeq
	10	*Broussonetia papyrifera, Poa pratensis*	0.0897, 0.9103	0.0160, 0.9840	GGC MiSeq
	11	*Broussonetia papyrifera, Poa pratensis*	0.0945, 0.9055	0.0169, 0.9831	GGC MiSeq

We extracted DNA from ~1,000,000 pollen grains for each sample; a quantity similar to what might be expected on the corbicula of a honeybee or a pooled sample from the bodies of multiple pollinating insects. The DNA isolation methods were described in full in Bell et al. (2019) and used the FastDNA™ Spin Kit for Soil (MP Biomedicals) with minor modifications as described in Bell et al. (2017). These DNA extractions were previously analyzed with DNA metabarcoding, based on *rbcL* and ITS2 (Bell et al., 2019). For *rbcL*, the primers rbcL2 (Palmieri et al., 2009) and rbcLa‐R (Kress & Erickson, 2007) were used. For ITS2, the primers ITS2 S2F and ITS2 S3R (Chen et al., 2010) were used. Illumina MiSeq libraries for DNA metabarcoding were prepared using Nextera XT dual‐index barcodes (Illumina) and run in a single flow cell on a 600‐cycle run of the MiSeq instrument at the Emory Integrated Genomics Core (EIGC). Taxonomic assignments were determined with the bioinformatics pipeline of Sickel et al. (2015), using the RDP classifier (Wang et al., 2007), and previously compiled and trained databases for ITS2 (Sickel et al., 2015) and *rbcL* (Bell et al., 2017), with the addition of relevant sequences that had become available more recently. For full details of the DNA metabarcoding analyses, see Bell et al. (2019).

### 2.3 Sample preparation and sequencing

We conducted whole‐genome shotgun sequencing of our samples in two groups, a first “pilot” group sequenced at the Emory Integrated Genomics Core (EIGC) on Illumina HiSeq and a second “full” group sequenced at the Georgia Genomics Facility (GGF) on Illumina MiSeq. While we recognize that ideally all sequencing would have been done in a single location with the same sequencing protocol and chemistry, logistical and financial constraints precluded this possibility. The first set of samples consisted of pollen mixtures 1 and 2 and single‐species *Carya illinoinensis* (Wangenh.) K. Koch (Juglandaceae), sequenced on an Illumina HiSeq at EIGC, with 2 M paired reads of 100 bp run for each sample from two flow cells, with library preparation using the Nextera XT method. Following successful results with the sample subset, we prepared mixtures 3–11 for sequencing by GGF using the Nextera XT library preparation method (Illumina). We also prepared single‐species samples (from the set of species included in the mixtures), as well as negative controls. Again, to ensure comparability of the WGS data with our earlier amplicon‐based study (Bell et al., 2019), we used the exact same isolated DNA. We incorporated a unique Illumina barcode combination to each DNA sample, so that all samples could be multiplexed. The multiplexed sample was run on Illumina MiSeq; a total of approximately 24–30 M paired reads of 150 bp was obtained for each multiplexed sample. This sampling strategy provided a similar quantity of data per sample as the HiSeq analysis, without the need to share flow cells with other projects. The samples run by EIGC (mix 1, mix 2, and *C*. *illinoinensis*) were run across two replicate flow cells. The samples run by GGF were run on a single flow cell. We analyzed data from the two replicate flow cells separately. These were treated as replicates in statistical analyses, so we had two replicate datasets for each sample run by EIGC and a single dataset for each sample run by GGF.

### 2.4 Bioinformatics

Taxonomic identification of WGS sequences was conducted using Kraken2 (Wood et al., 2019). This program compares short sequence substrings (*k*‐mers) from the sample sequences to a genomic reference database, returning the lowest common ancestor of matches in the database. A Kraken2 (Wood et al., 2019) database was created (March, 2020) using all plant assemblies available in the NCBI RefSeq database (O’Leary et al., 2016). Additional plant species were included from GenBank (Clark et al., 2016) and the Sequence Read Archive (Leinonen et al., 2011) (SRA), to ensure that all species in pollen mixtures were represented (Table 3). Genome assemblies were downloaded from GenBank with ncbi‐genome‐download (Blin, n.d.) (v0.2.12). Raw FASTQs for six species were downloaded from SRA with fasterq‐dump (NCBI, n.d.) (v2.10.0) and converted to FASTA with reformat.sh (Bushnell, 2016) (v38.75). For SRA projects with more than 10 Runs, only 10 randomly selected Runs were downloaded to control file sizes. All downloaded FASTAs were formatted for Kraken2 and added to the RefSeq plant database.

**TABLE 3:** NCBI data for taxa of interest included in our kraken database, in addition to all available RefSeq assemblies

Species	Taxon ID	Accession	Type	References
*Ambrosia artemisiifolia*	4212	PRJNA449949	FASTQ	(van Boheemen et al., 2017)
*Artemisia tridentata*	55611	PRJNA258169	FASTQ	(Huynh et al., 2015)
*Bassia scoparia*	83154	GCA_008642245	Assembly	(Patterson et al., 2019)
*Broussonetia papyrifera*	172644	PRJNA437223	FASTQ	(Peng et al., 2019)
*Carya illinoinensis*	32201	GCA_011037805	Assembly	(Huang et al., 2019)
*Poa pratensis*	4545	PRJNA517968	FASTQ	(Y. Chen et al., 2019)
*Populus deltoides*	3696	PRJNA430966	FASTQ	(Zhu et al., 2018)
*Populus tremuloides*	3693	PRJNA299390	FASTQ	No linked publication

Raw reads sequenced from this study underwent error correction and quality filtering using the Nextflow (Di Tommaso et al., 2017) (v19.10) workflow illumina‐cleanup (Petit, n.d.‐b) (v1.0.0) with the default settings. A file of filenames was generated using the Bactopia (Petit & Read, 2020) prepare function and was used as input for Illumina‐cleanup. Reads were trimmed and quality‐filtered with bbduk.sh (Bushnell, 2016) (v38.75) and error‐corrected with Lighter (Song et al., 2014) (v1.1.1). Sequence quality metrics were created with fastq‐scan (Petit, n.d.‐a) and FastQC (Andrews et al., 2016). The processed FASTQs were queried against the custom Kraken2 database.

The tools used in this analysis are each available from Bioconda (Grüning et al., 2018). The commands and results are available at https://github.com/Brosi‐Lab/Kraken.

### 2.5 Data analysis

#### 2.5.1 Overview and analysis commonalities

Our analysis focused on three outcomes: 1) false‐negative identifications, that is, failure to identify a taxon that was present in a sample; 2) false‐positive identifications, that is, incorrect identification of taxa not present in a sample; and 3) quantitative matching, that is, the relationship between the proportion of pollen grains in a sample and the proportion of sequence counts corresponding to that taxon in our output. For each of these outcomes, we assessed WGS on its own and separately compared the performance of WGS with our previous amplicon results (again from the exact same DNA extractions). For the qualitative outcomes (false negatives and positives), we assessed the effect of sample complexity on matching, including the constructed pollen mixtures described above, specifically constructed to vary in species richness, relatedness of plant taxa, and rarity.

We conducted all statistical analyses in the R language for statistical programming (R Core Team, 2016), specifically using Rmarkdown in the RStudio platform. A fully reproducible Rmarkdown file of our analyses is included in the GitHub repository at https://github.com/Brosi‐Lab/Kraken. To enable comparison to amplicon results, we removed identifications to *Zea mays* L. (Poaceae) throughout our analyses. We excluded this taxon from amplicon analyses to allow comparison with DNA metabarcoding, because it did not amplify with ITS2 and was not identifiable to genus with *rbcL* (Bell et al., 2019). We excluded mixture 5, containing *Populus deltoides* W. Bartram ex Marshall (Salicaceae) and *Populus tremuloides* Michx. (Salicaceae), as we could not be certain of the actual species proportions since these species could not be differentiated by microscopy. Because all analyses included nonindependent data (multiple replicates of the same pollen mixtures; pollen from the same plant species occurring in multiple mixtures), all our analyses were conducted with mixed‐effects modeling, using mixture identity and species identity as crossed random effects (modeled as random intercepts). Across our outcomes, in comparing WGS and amplicon performance, we pooled WGS and amplicon results together into a single data table and conducted analyses with sequencing method (WGS vs. amplicon) as a fixed effect.

#### 2.5.2 Contaminant sequence removal

We removed sequences indistinguishable from sample contamination using a criterion based on our negative controls. Specifically, we removed Kraken2‐based taxonomic assignments recorded from fewer sequence reads than for the highest number obtained from any negative control (DNA isolation and sequencing negative controls) as such reads cannot be distinguished from sample contamination. This was a conservative criterion, as the normalization of samples prior to sequencing meant that negative control samples were added to the library pool at higher volumes than pollen DNA samples.

#### 2.5.3 Outcomes 1 and 2: false negatives and false positives

The response variables for our first two outcomes were binomial (yes / no) in structure. For our first outcome of false negatives, we needed to record—for each species present in a pollen mixture—whether or not that species was identified in that sample. To do this, we set up a data file with each species truly present within each sample as its own row, which we subsequently scored as 0 / 1, with a zero for species that were present in the sample but not identified in sequencing reads above the contamination threshold, and a one for species that were identified in the sequencing reads above the threshold. For our second outcome of false positives, we assessed the proportion of true vs. false positives. To do this, we aggregated the data to one row per sample replicate and summed the read counts of true positives (combining counts of all species truly present in a particular mix) and false positives in two separate columns.

We first asked how WGS performed on its own in terms of false‐negative and false‐positive reads, specifically in terms of how sample complexity affected these outcomes. We tested the effect of three forms of sample complexity on the ability to detect the presence or absence of a species in a mixture, based on pollen mixtures we designed: (1) species richness of the sample; (2) taxonomic relatedness within the sample (0 = same species; 1 = same genus; 2 = same family; 3 = same class; 4 = different classes); and (3) rarity of the species (proportion of pollen grains in a sample). We separately analyzed positive matches at two taxonomic levels: genus and species. We ran separate binomial‐errors mixed‐effects models for each of these three questions of interest (species richness, taxonomic relatedness, and rarity) with each of those variables included as the sole fixed effect in that respective model. Subsequently, we pooled both the amplicon and the WGS data into new combined data files, and conducted analyses of both false‐negative and false‐positive matching using method (DNA metabarcoding vs. WGS) as the sole fixed effect, providing a direct comparison of the performance of our WGS method to metabarcoding methods.

#### 2.5.4 Quantitative accuracy

To assess the quantitative accuracy of WGS sequencing for our constructed mixtures, we tested the correlation between the (known) proportion of pollen grains in a sample (Bell et al., 2019) and the proportion of DNA sequencing reads (i.e., the proportion of reads assigned to a taxon at the taxonomic level of interest relative to the total classified sequencing reads). We used a linear mixed‐effects model, implemented with the “lmerTest” package in R. The response variable was the proportion of sequencing reads, and the explanatory variable was proportion of input pollen grains. In parallel with our qualitative analyses, we used mixture identity and species as crossed random‐intercepts effects. This analysis was conducted separately for identifications at the level of species, genus, and family.

## 3 RESULTS

### 3.1 Overview

For the three samples run on HiSeq, we obtained 4,188,389 paired‐end sequencing reads of 101bp, ranging from 645,680 to 765,835 reads per sample per flow cell (Table 4). Of these, 679,753 to 1,279,514 were retained per sample after combining data from both flow cells, quality filtering, and classification. For the remaining samples run on MiSeq, we obtained 13,515,962 total sequencing reads of 150 bp, ranging from 647,261 to 1,009,158 per sample, excluding the *Broussonetia papyrifera* (L.) Vent. sample where only 7 sequencing reads were obtained. Of these, 447,215 to 839,951 were retained per sample after quality filtering and classification. Across both sets of samples, Kraken2 identified 11,984,584 reads (92.7% of classified reads) to the level of species and 12,644,534 reads (97.8%) to at least the level of genus. This compares to 34.5% of reads identified to species and 57.6% to genus with *rbcL* amplicon sequencing and 47.9% to species and 83.4% to genus with ITS2 amplicon sequencing (Bell et al., 2019). Negative control samples were all run on the MiSeq platform and yielded 507 and 6,621 sequencing reads per sample. Of these, 333 and 5,048 were retained per sample after quality filtering and classification. Therefore, a contaminant threshold of 5,048 was used for retaining taxonomic identifications in samples.

**TABLE 4:** Number of sequencing reads and Kraken2 k‐mer fragments retained for analysis WGS sequencing data of pollen samples through processing steps of quality filtering and classification

Sample	Total reads	Retained after filtering	Classified Fragments	Unclassified Fragments	Level of classification	
					Genus	Species
Mixture 1	2,737,806	2,418,106	706,013 (58.39%)	503,040 (41.61%)	680,193 (56.30%)	669,890 (55.45%)
Mixture 2	2,589,970	2,163,054	679,753 (62.85%)	401,774 (37.15%)	660,046 (61.04%)	653,062 (60.38%)
Mixture 3	1,832,234	1,715,760	639,808 (74.58%)	218,072 (25.42%)	624,267 (72.76%)	603,821 (70.38%)
Mixture 4	1,877,360	1,795,644	682,921 (76.06%)	214,901 (23.94%)	665,730 (74.19%)	635,180 (70.77%)
Mixture 5	1,583,926	1,498,748	744,876 (99.40%)	4,498 (0.60%)	731,490 (97.55%)	563,733 (75.17%)
Mixture 6	1,524,128	1,381,686	525,819 (76.11%)	165,024 (23.89%)	516,610 (74.78%)	513,957 (74.38%)
Mixture 7	1,841,202	1,777,146	581,375 (65.43%)	307,198 (34.57%)	560,525 (63.05%)	550,258 (61.89%)
Mixture 8	1,499,692	1,421,518	543,432 (76.46%)	167,327 (23.54%)	533,301 (75.04%)	530,254 (74.61%)
Mixture 9	1,294,522	1,227,674	458,933 (74.76%)	154,904 (25.24%)	450,772 (73.42%)	448,198 (73.01%)
Mixture 10	1,355,242	1,208,520	447,215 (74.01%)	157,045 (25.99%)	439,119 (72.64%)	436,536 (72.21%)
Mixture 11	2,018,316	1,828,026	673,577 (73.69%)	240,436 (26.31%)	661,246 (72.33%)	657,267 (71.89%)
*Ambrosia artemisiifolia*	1,924,176	1,877,500	656,579 (69.94%)	282,171 (30.06%)	630,876 (67.23%)	621,140 (66.16%)
*Artemisia tridentata*	1,770,424	1,722,090	554,707 (64.42%)	306,338 (35.58%)	534,603 (62.09%)	524,307 (60.90%)
*Bassia scoparia*	1,790,256	1,735,430	839,951 (96.80%)	27,764 (3.20%)	832,989 (96.00%)	831,720 (95.84%)
*Broussonetia papyrifera*	30	14	5 (71.43%)	2 (28.57%)	5 (71.43%)	5 (71.43%)
*Carya illinoinensis*	3,049,002	2,687,214	1,279,514 (95.23%)	64,093 (4.77%)	1253842 (93.29%)	1,250,232 (92.97%)
*Poa pratensis*	1,817,984	1,736,554	640,084 (73.72%)	228,193 (26.28%)	628302 (72.35%)	624,364 (71.89%)
*Populus deltoides*	1,613,062	1,565,038	773,325 (98.83%)	9,194 (1.17%)	758913 (96.94%)	556,430 (71.07%)
*Populus tremuloides*	1,664,188	1,613,334	804,651 (99.75%)	2,016 (0.25%)	791915 (98.11%)	624,746 (77.39%)
*Zea mays*	1,610,926	1,438,576	700,507 (97.39%)	18,781 (2.61%)	689128 (95.76%)	688,837 (95.68%)
Negative control 1	13,242	10,096	152 (45.65%)	181 (54.35%)	141 (42.34%)	128 (38.43%)

### 3.2 Taxonomic Identifications

#### 3.2.1 False negatives

**FIGURE 1:**
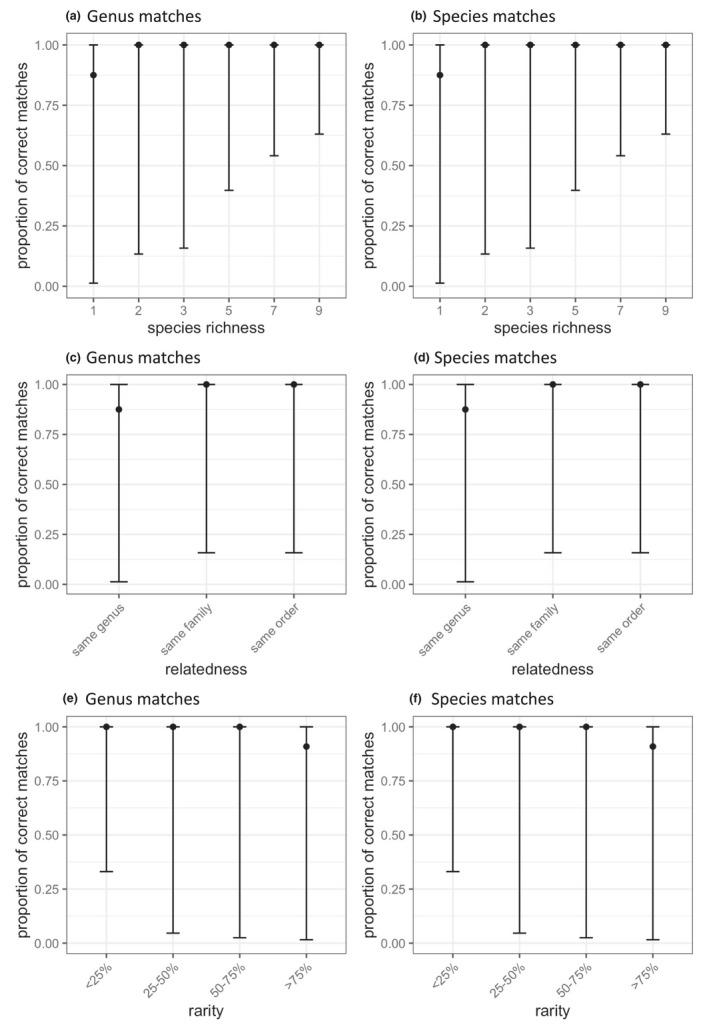
Proportion of samples with correct matches at each taxonomic level, as samples vary in complexity. (a, b) mixtures of varying species richness; (a) genus‐level identifications; (b) species‐level identifications. (c, d) mixtures containing pairs of taxa of varying relatedness; (c) genus‐level identifications; (d) species‐level identifications. (e, f) mixtures of two species varying in proportion of the rarest species; (e) genus‐level identifications; (f) species‐level identifications

A list of the species identified from at least 1% of sequencing reads in each sample is available in Appendix S1. In all mixtures except for the sample containing *B*. *papyrifera* alone, Kraken2 analysis of WGS identified all taxa in the mixture to the species level (Table 5; Figure 1). The sample containing *B*. *papyrifera* alone contained only 7 reads, which was below the contamination threshold. We were unable to conduct mixed‐effects modeling of the effect of sample complexity on true positive detection, due to the almost 100% success rate.

**TABLE 5:** Qualitative accuracy of WGS of constructed pollen mixtures, in terms of correct species, genus and family level identification. For complete lists of taxa and numbers of sequencing reads, see Supplementary information s2 and s3

Mixture	Species	True positive identification	
		Genus	Species
1	*Broussonetia papyrifera*	Yes	Yes
	*Artemisia tridentata*	Yes	Yes
	*Zea mays*	Yes	Yes
2	*Broussonetia papyrifera*	Yes	Yes
	*Bassia scoparia*	Yes	Yes
	*Artemisia tridentata*	Yes	Yes
	*Poa pratensis*	Yes	Yes
	*Zea mays*	Yes	Yes
3	*Populus tremuloides*	Yes	Yes
	*Broussonetia papyrifera*	Yes	Yes
	*Carya illinoinensis*	Yes	Yes
	*Bassia scoparia*	Yes	Yes
	*Artemisia tridentata*	Yes	Yes
	*Poa pratensis*	Yes	Yes
	*Zea mays*	Yes	Yes
4	*Populus tremuloides*	Yes	Yes
	*Populus deltoides*	Yes	Yes
	*Broussonetia papyrifera*	Yes	Yes
	*Carya illinoinensis*	Yes	Yes
	*Bassia scoparia*	Yes	Yes
	*Ambrosia artemisiifolia*	Yes	Yes
	*Artemisia tridentata*	Yes	Yes
	*Poa pratensis*	Yes	Yes
	*Zea mays*	Yes	Yes
5	*Populus tremuloides*	Yes	Yes
	*Populus deltoides*	Yes	Yes
6	*Poa pratensis*	Yes	Yes
	*Zea mays*	Yes	Yes
7	*Broussonetia papyrifera*	Yes	Yes
	*Artemisia tridentata*	Yes	Yes
8	*Broussonetia papyrifera*	Yes	Yes
	*Poa pratensis*	Yes	Yes
9	*Broussonetia papyrifera*	Yes	Yes
	*Poa pratensis*	Yes	Yes
10	*Broussonetia papyrifera*	Yes	Yes
	*Poa pratensis*	Yes	Yes
11	*Broussonetia papyrifera*	Yes	Yes
	*Poa pratensis*	Yes	Yes
Single species	*Populus tremuloides*	Yes	Yes
	*Populus deltoides*	Yes	Yes
	*Broussonetia papyrifera*	No	No
	*Carya illinoinensis*	Yes	Yes
	*Bassia scoparia*	Yes	Yes
	*Ambrosia artemisiifolia*	Yes	Yes
	*Artemisia tridentata*	Yes	Yes
	*Poa pratensis*	Yes	Yes
	*Zea mays*	Yes	Yes

Whether WGS using our full or partial genome reference database was more or less effective than amplicon sequencing using the more complete locus‐specific barcode reference databases for the detection and identification of pollen taxa in a mixture depended on the taxonomic level of identification and the barcode used for DNA metabarcoding (Table 6). In comparison to either *rbcL* or ITS2 alone, the WGS method identified significantly more taxa correctly at both the species and the genus level. After combining *rbcL* and ITS2 identifications, DNA metabarcoding still performed significantly worse than WGS for the identification of taxa at the species levels, but there was no significant difference at the genus level.

**TABLE 6:** Binomial mixed model to assess if method (WGS or amplicon sequencing) has a significant effect on the ability to identify true positives in a pollen mixture

DNA barcode	Taxonomic level	*p*‐value	*N*
*rbcL*	Species	.0000003	299
	Genus	.0209759	299
ITS2	Species	.0001272	299
	Genus	.0002302	299
*rbcL* and ITS2 combined	Species	.0027829	294
	Genus	.4333710	294

#### 3.2.2 False positives

**FIGURE 2:**
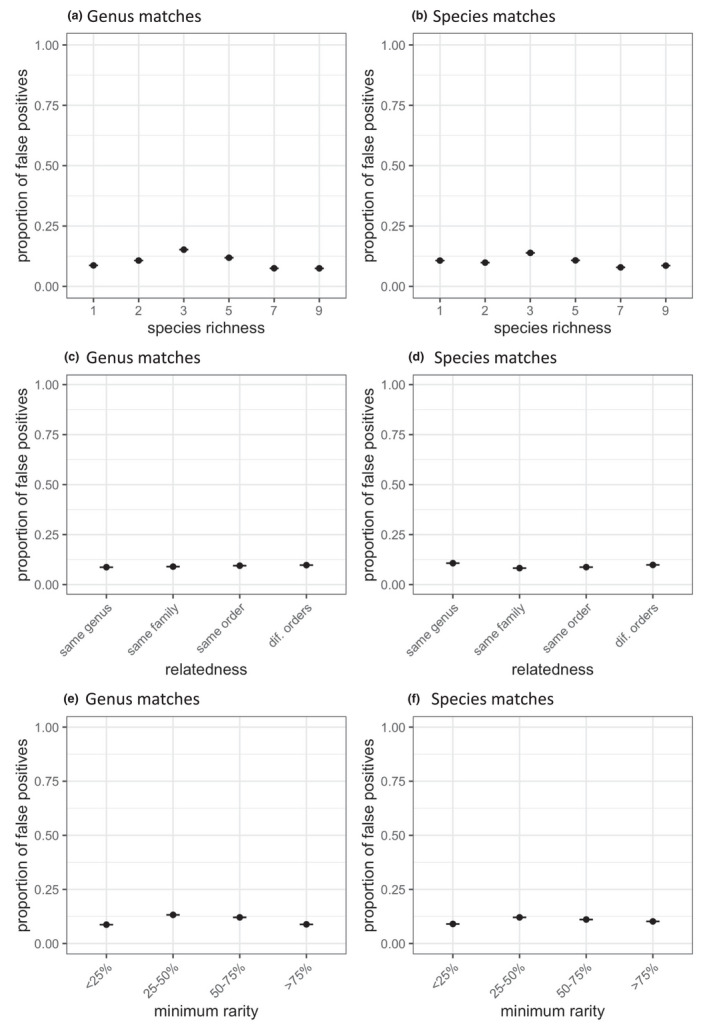
Proportion of false‐positive sequencing reads at each taxonomic level, as samples vary in complexity. (a, b) mixtures of varying species richness; (a) genus‐level identifications; (b) species‐level identifications. (c, d) mixtures containing pairs of taxa of varying relatedness; (c) genus‐level identifications; (d) species‐level identifications. (e, f) mixtures of two species varying in proportion of the rarest species; (e) genus‐level identifications; (f) species‐level identifications

False positives occurred in all samples (Appendix S1). Species‐level false‐positive identifications usually occurred as less than 1% of the total reads in the sample. Excluding the *Broussonetia papyrifera* single‐species sample, which had very few sequencing reads, only three false‐positive species were identified from greater than 1% of the total reads. *Helianthus annuus* L. was identified in the *Ambrosia artemisiifolia* L. single‐species sample at 17.08% of total reads, the *Artemisia tridentata* Nutt. single‐species sample at 2.33% of total reads, and mixtures containing these two species at up to 2.43% of total reads. *Populus tremuloides* Michx. identifications occurred in the single‐species samples of *Populus deltoides* W. Bartram ex Marshall at 7.68%, and several other single‐species samples and mixtures at up to 2.6%. *P*. *deltoides* was identified in the single‐species *P*. *tremuloides* sample at 3.17% of total reads. We found no evidence that sample complexity (relatedness, species richness, and pollen grain proportion) affected the proportion of false‐positive identifications at the species or genus level (Table 7; Figure 2).

**TABLE 7:** Binomial mixed‐effects model to assess if species richness, species relatedness, and pollen grain proportion have a significant effect on the proportion of false‐positive sequencing reads identified in a pollen mixture through WGS analyzed with kraken

Measure of mixture complexity	Taxonomic level	*p*‐value	n
Species richness	Species	.8957640	17
	Genus	.3812160	17
Taxonomic relatedness	Species	.5412578	17
	Genus	.0984720	17
Pollen grain proportion	Species	N/A^a^	17
	Genus	N/A	17

^a^ Model did not converge.

Comparing WGS to amplicon sequencing, we found that the two approaches did not produce significantly different rates of false‐positive reads at the species level, irrespective of the marker used (*rbcL*, ITS2, or both markers combined) (Table 8). At the genus level, ITS2 amplicon sequencing generated significantly fewer false positives than WGS. When the two amplicon markers were combined, there was no significant difference in false‐positive rate between amplicons and WGS.

**TABLE 8:** Binomial mixed model to assess if method (WGS or amplicon sequencing) has a significant effect on the proportion of false‐positive sequencing reads identified in a pollen mixture. The direction of the trend was always in favor of amplicon sequencing

DNA barcode	Taxonomic level	*p*‐value	N
*rbcL*	Species	.5067176	54
	Genus	.6917513	54
ITS2	Species	.9102740	54
	Genus	.0000454	54
*rbcL* and ITS2 combined	Species	.9694023	106
	Genus	.0993345	106

### 3.3 Quantitative matching

**FIGURE 3:**
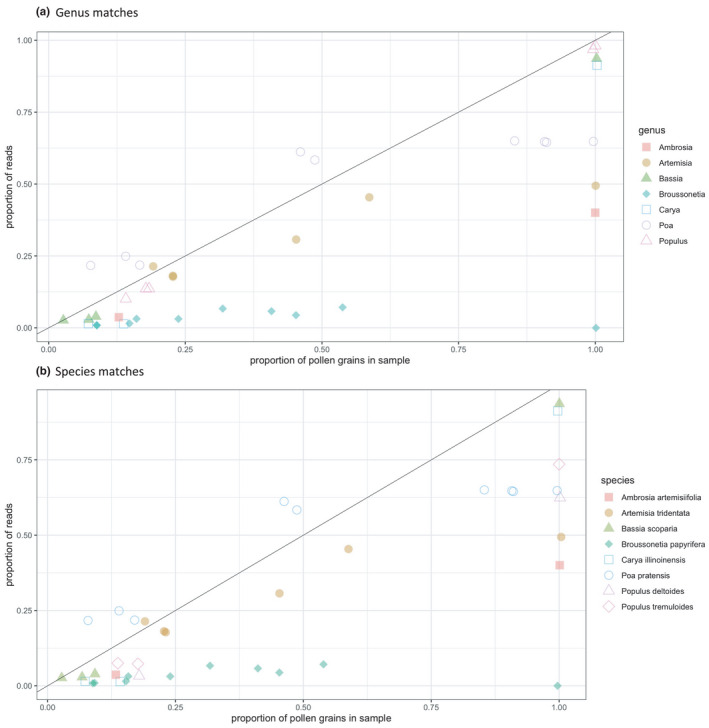
Relationship between the proportion of pollen grains in a mixture belonging to a particular taxon and the proportion of whole‐genome shotgun sequencing reads matching that taxon: (a) genus‐level identifications; and (b) species‐level identifications

The proportion of WGS sequencing reads identified to a particular taxon was usually less than the proportion of pollen grains of that taxon in the mixture (Figure 3), as is expected given the presence of false‐positive reads. We found that the proportion of DNA sequencing reads for each taxon increased with an increasing proportion of pollen grains within a mixture at the genus (*R^2^* = .62, *p* < .000001) and species (*R^2^* = .60, *p* = .0007) levels, but the slope of the regression was well below 1 (0.46 for both genus and species).

Quantitatively, WGS provided a better fit than amplicons in terms of the strength of correlation between pollen grain proportion and read proportion, regardless of the barcode or taxonomic rank (WGS results in the preceding paragraph; for *rbcL* slope = 0.55, *R^2^* = .40 at genus level, slope =0.39, *R^2^* = .30 at species level; for ITS2 slope = 0.48, *R^2^* = .26 at genus level, slope = 0.24, *R^2^* = .09 at species level). WGS data, however, departed more strongly from a 1:1 proportional pollen grain input to sequence read output relationship (greater residuals) relative to amplicon data, with this difference being statistically significant compared to *rbcL* at the genus level (differing by a factor of −0.061, *p* = .025) but not the species level (−0.023, *p* = .474), and ITS2 at the species level (−0.068, *p* = .003) but not the genus level (−0.027, *p* = .103). This discrepancy from the previous result was likely driven by the higher proportion of false‐positive reads in the WGS data.

## 4 DISCUSSION

We tested the ability of WGS to identify taxa and quantify their relative abundances in pollen mock communities, using ~600,000–700,000 sequencing reads per sample and Kraken2‐based taxonomic identification with publicly available reference sequences and compared this to our previous DNA metabarcoding analyses. We found our WGS method to be almost 100% successful in identifying known species in mixtures. Rates of false negatives (failure to detect and identify taxa that were present) and false positives (reads that were not identified as taxa that were present) in WGS data were not sensitive to mixture complexity. In comparison to DNA metabarcoding, WGS performed better in terms of false negatives and worse in terms of false positives. We found WGS to give improved quantification of the proportions of taxa when compared to metabarcoding, although the relationship between input pollen grain proportion and read count proportions deviates from the expected 1:1 relationship.

### 4.1 Species‐level identification

Compared to DNA metabarcoding, our WGS sequencing method generated fewer false negatives. WGS generated more false‐positive reads, though this difference was not statistically significant for most comparisons. There have been few studies assessing the detection and identification ability of WGS relative to amplicon sequencing, and most of these are not directly comparable to our study due to differences in experimental design. Bista et al. (2018) found better qualitative detection with WGS compared to amplicon sequencing of COI for mixtures of invertebrate animals. However, their study used mitochondrial data only (<1% of sequencing reads), and their reference library was custom‐made for their study using only the species of interest and assembling the full mitochondrion. In contrast, our study uses nuclear, chloroplast, and mitochondrial genomes, with a reference database of all available RefSeq genomes. Other studies using WGS methods for pollen identification have also found that the ability to identify all species in a mixture is close to 100% (Lang et al., 2019; Peel et al., 2019). It is not possible to compare our false‐positive rate to either of these studies based on differences in the way identifications to taxonomic levels other than species or genus are treated (i.e., removed vs treated as false positives). It is likely that our false‐positive rate is similar to these studies as there were few false‐positive species‐level identifications occurring at greater than 1% in any sample.

Differences in the false‐positive rate between WGS and DNA metabarcoding could be explained by the relative quality and completeness of the reference databases. A recent study aiming to analyze plant diet from fecal samples encountered a high false‐positive rate using whole‐genome reference sequences due to missing species, which limited the use of WGS data to a few well‐represented loci (Chua et al., 2021). Although all the species included in our mixtures were present in our reference database for WGS, many were downloaded from NCBI as unassembled SRA data and may have had lower coverage and more errors than RefSeq genome assemblies. Genome assembly data may contain contaminants (e.g., fungi, bacteria, human; Breitwieser et al., 2019) that would not amplify with the kingdom‐specific primers typically used for DNA metabarcoding, but would affect taxonomic matching for WGS methods. As more genome sequence data become available and reference genome quality improves, false‐negative and false‐positive reads in WGS are likely to diminish, as has been recorded for bacterial metagenomics (Nasko et al., 2018).

### 4.2 Quantification of relative species abundances

Several studies have found the relationship between DNA metabarcoding sequence reads and pollen proportion to deviate substantially from a 1:1 relationship, especially with ITS2 (Bell et al., 2019; Richardson, Lin, Sponsler, et al., 2015; Smart et al., 2017). Using WGS, we detected a stronger correlation between the proportion of pollen grains of a taxon in a mixture and the proportion of sequences assigned to that taxon, relative to DNA metabarcoding. At the same time, we also found that the WGS sequence reads departed more strongly from the “true” 1:1 input pollen grain to output sequence proportion relationship relative to amplicon‐based methods. Comparison of WGS of chloroplast genomes with DNA metabarcoding for the identification of pollen mixtures has found improved quantification with WGS (Lang et al., 2019), and likewise for comparison of WGS of mitochondrial genomes with amplicon sequencing of animal mixtures (Bista et al., 2018), and this quantification can be further improved by correcting for mitochondrial genome copy number (Garrido‐Sanz et al., 2020; Garrido‐Sanz et al., 2021). Long‐read sequencing of nuclear DNA of pollen mixtures has been found to be semiquantitative, in that species present in high proportions were detected in high proportions and species present in low proportions were detected in low proportions (Peel et al., 2019). Our WGS short‐read sequencing could be similarly described as semiquantitative.

There are at least three reasons why WGS may only be semiquantitative. First, pollen grains vary in their DNA quantity due to variation in genome size, which can differ by orders of magnitude among flowering plant species (Soltis et al., 2003). Kraken2 was not designed for quantification and does not correct for genome size bias, so some of the unexplained variance in our analyses may be due to this. This could be corrected with knowledge of genome sizes, though variation in ploidy within taxa (Kolář et al., 2017) could complicate such corrections. Second, the proportion of sequencing reads identified to a taxon was always less than the true proportion of pollen grains for that taxon in the sample due to false positives. Improved coverage and quality of whole‐genome reference databases is likely to reduce the false‐positive rate and improve quantification in the future. Third, as with amplicon sequencing, our DNA extractions may have been affected by variation among species in the effectiveness of the extraction. This source of bias could be corrected with a database of relative DNA extraction efficiencies.

### 4.3 Present feasibility of WGS and future research directions

Our results highlight the potential of WGS as a method for identification and quantification of pollen in mixtures. Based on the current state of technology and reference databases, WGS provides an improvement in quantification, but with a higher rate of false positives. Currently, we see three disadvantages of WGS over DNA metabarcoding, although solutions may be provided in the near future. First, WGS methods are only suitable for study systems where reference genomes are available for the majority of species. Globally, the number of plant species with full‐genome sequences in public databases is much smaller than the number of species with *rbcL* or ITS2 sequence. Our WGS reference database (assembled in March, 2020) included 93 flowering plant species with RefSeq genomes, 2 species with GenBank assemblies, and 6 species represented by SRA data (WGS or RNA‐seq). The reference databases used in our DNA metabarcoding study (assembled January 27, 2016, and January 19, 2015, respectively) included 38,409 species with *rbcL* sequence and 72,325 species with ITS2 sequence. This compares to an estimated 450,000 flowering plant species on the planet (Pimm & Joppa, 2015). Without substantial increases in the number of publicly available reference sequences, the power of WGS will be limited by the need to generate reference genomes prior to conducting taxonomic identification. Second, sequencing costs are higher for WGS than DNA metabarcoding. We used one 600 cycle run of Illumina MiSeq for 96 samples in the DNA metabarcoding study (Bell et al., 2019) and one 300 cycle run of Illumina MiSeq for 22 samples in the current study. This represents an approximately 4.5‐fold increase in per sample sequencing costs for WGS. However, sequencing costs are likely to decrease over time, while other costs such as fieldwork and staff time, are likely to increase, making the sequencing cost less important in selecting the most appropriate method. Currently, the biggest cost differential is likely to be in the preparation of reference databases because there are currently more species with publicly available barcode reference sequences than genomic reference sequences. Generating new reference genomes would incur costs from fieldwork, laboratory analyses, bioinformatics, storage of sequence data, and deposition of herbarium specimens. Third, a higher quality and quantity of DNA is required for WGS. In this study, we based our analysis on DNA extractions of samples containing 1,000,000 pollen grains. A similarly high number of pollen grains is likely to be achievable for pollen loads from the corbiculae of bees, but pollen samples recovered from the bodies of pollinating insects, particularly those of small body size, are likely to have much fewer pollen grains and would need to be pooled before DNA extraction. Improvements in library preparation kits mean that required DNA quantities are decreasing. The most recent version of the Illumina Nextera XT DNA Library Preparation Kit requires only 1 ng of input DNA. This quantity would be achievable from pollen loads from individual pollinators.

These three disadvantages are likely to be minimized with future developments. Currently, the most important requirement to make WGS identification of pollen mixtures feasible is improved reference sequence databases. While the current number of publicly available high‐quality genome assemblies represent only a small proportion of plant species diversity, it is likely that sequencing rates will increase with new initiatives such as the Earth BioGenome Project, which aims to have sequenced the genomes of most eukaryote species within a decade (Lewin et al., 2018). New methods using long sequencing reads and newly developed bioinformatics pipelines will increase the rate at which plant genomes can be assembled (Driguez et al., 2021). Database “cleaning” will be possible when there are more near‐complete genomes from a wider range of species, and new bioinformatics methods for removing bacterial contaminants from eukaryotic genome assemblies show promise (Fierst & Murdock, 2017). With future increases in availability and quality of reference genome sequences, WGS will become feasible for the identification and quantification of pollen in most applications.

## 5 CONCLUSIONS

The limitations of DNA metabarcoding mean that alternatives need to be developed. We have demonstrated that WGS is a suitable method for identification and quantification of pollen grains in mixtures, although it may not currently be practical. The weaknesses of WGS are surmountable in the long‐term, particularly as the number of publicly available full‐genome sequences increases. Increased reference sequence availability will enable WGS to identify species (or taxa below the species level) that are not uniquely identifiable via DNA barcoding and allow for improved quantification of the proportions of species in a mixture. This higher level of precision would allow for finer geographic precision in forensic applications, improved understanding of pollination biology at the plant population level, and more accurate assessments of food origins and quality. We anticipate that genomics methods will become the methods of choice for identification of pollen and other plant mixtures within the next decade.

## CONFLICT OF INTEREST

None declared.sss

## AUTHOR CONTRIBUTION

**Karen L. Bell:** Conceptualization (supporting); Data curation (lead); Formal analysis (supporting); Methodology (lead); Project administration (supporting); Writing‐original draft (lead). **Robert A. Petit:** Formal analysis (lead). **Anya Cutler:** Formal analysis (supporting). **Emily K. Dobbs:** Formal analysis (supporting); Methodology (supporting); Project administration (supporting). **J. Michael Macpherson:** Formal analysis (supporting). **Timothy D. Read:** Conceptualization (equal); Formal analysis (supporting); Funding acquisition (supporting); Methodology (supporting); Supervision (supporting); Writing‐review & editing (equal). **Kevin S. Burgess:** Conceptualization (supporting); Funding acquisition (supporting); Methodology (supporting); Supervision (supporting); Writing‐review & editing (equal). **Berry J. Brosi:** Conceptualization (lead); Data curation (supporting); Formal analysis (supporting); Funding acquisition (lead); Methodology (supporting); Project administration (lead); Supervision (lead); Writing‐review & editing (equal).

## Supporting information

Supplementary Material

## Data Availability

Our analysis pipeline can be found at https://github.com/Brosi‐Lab/Kraken. Sequence data have been deposited as a SRA on the NCBI database under BioProject ID PRJNA542384 (https://www.ncbi.nlm.nih.gov/bioproject/PRJNA542384).
