# Supplementary material for: Comparing whole‐genome shotgun sequencing and DNA metabarcoding approaches for species identification and quantification of pollen species mixtures
Source: Ecol Evol. 2021 Nov 4;11(22):16082–98. doi: 10.1002/ece3.8281 (PMC8601920; doi:10.1002/ece3.8281)
Supplement: Supplementary file 1 — Supplementary Material [file ECE3-11-16082-s001.docx]

**Appendix 1:** Taxonomic identifications of WGS of pollen mixtures classified by Kraken 2. Taxa represented by fewer than 1% of reads in a sample are not shown.

**Mixture 1**

| Percentage of reads within taxon | Number of reads within taxon | Number of reads identified as taxon | Taxonomic rank | NCBI taxon ID | Taxon |
| --- | --- | --- | --- | --- | --- |
| 41.61 | 503040 | 503040 | U | 0 | unclassified |
| 58.39 | 706013 | 0 | R | 1 | root |
| 58.39 | 706013 | 0 | R1 | 131567 | cellular organisms |
| 58.39 | 706013 | 37 | D | 2759 | Eukaryota |
| 58.39 | 705976 | 49 | K | 33090 | Viridiplantae |
| 58.39 | 705917 | 0 | P | 35493 | Streptophyta |
| 58.39 | 705917 | 0 | P1 | 131221 | Streptophytina |
| 58.39 | 705917 | 183 | P2 | 3193 | Embryophyta |
| 58.33 | 705296 | 0 | P3 | 58023 | Tracheophyta |
| 58.33 | 705296 | 0 | P4 | 78536 | Euphyllophyta |
| 58.33 | 705296 | 0 | P5 | 58024 | Spermatophyta |
| 58.33 | 705296 | 392 | C | 3398 | Magnoliopsida |
| 58.27 | 704571 | 5119 | C1 | 1437183 | Mesangiospermae |
| 46.62 | 563658 | 0 | C2 | 71240 | eudicotyledons |
| 46.62 | 563658 | 0 | C3 | 91827 | Gunneridae |
| 46.62 | 563658 | 7645 | C4 | 1437201 | Pentapetalae |
| 33.89 | 409759 | 1174 | C5 | 71274 | asterids |
| 32.72 | 395613 | 193 | C6 | 91882 | campanulids |
| 32.62 | 394439 | 0 | O | 4209 | Asterales |
| 32.62 | 394439 | 1104 | O1 | 4210 | Asteraceae |
| 32.07 | 387703 | 650 | O2 | 102804 | Asteroideae |
| 30.7 | 371121 | 0 | O3 | 102810 | Anthemideae |
| 30.7 | 371121 | 0 | O4 | 886714 | Artemisiinae |
| 30.7 | 371121 | 0 | G | 4219 | Artemisia |
| 30.7 | 371121 | 371121 | S | 55611 | Artemisia tridentata |
| 1.32 | 15932 | 0 | O3 | 911341 | Heliantheae alliance |
| 1.32 | 15932 | 169 | O4 | 102814 | Heliantheae |
| 1.26 | 15202 | 0 | G | 4231 | Helianthus |
| 1.26 | 15202 | 15202 | S | 4232 | Helianthus annuus |
| 1.07 | 12972 | 123 | C6 | 91888 | lamiids |
| 11.86 | 143348 | 1272 | C5 | 71275 | rosids |
| 11.03 | 133330 | 3858 | C6 | 91835 | fabids |
| 7.01 | 84752 | 243 | O | 3744 | Rosales |
| 6.69 | 80929 | 0 | O1 | 3487 | Moraceae |
| 6.69 | 80929 | 0 | G | 66379 | Broussonetia |
| 6.69 | 80929 | 80929 | S | 172644 | Broussonetia papyrifera |
| 2.28 | 27595 | 45 | O | 3646 | Malpighiales |
| 2.22 | 26891 | 0 | O1 | 3688 | Salicaceae |
| 2.22 | 26891 | 0 | O2 | 238069 | Saliceae |
| 2.22 | 26891 | 2743 | G | 3689 | Populus |
| 1.42 | 17200 | 17200 | S | 3693 | Populus tremuloides |
| 1.11 | 13404 | 0 | O | 72025 | Fabales |
| 1.11 | 13404 | 0 | O1 | 3803 | Fabaceae |
| 1.11 | 13404 | 0 | O2 | 3814 | Papilionoideae |
| 1.11 | 13404 | 305 | O3 | 2231393 | 50 kb inversion clade |
| 11.08 | 133996 | 0 | C2 | 4447 | Liliopsida |
| 11.08 | 133996 | 50 | C3 | 1437197 | Petrosaviidae |
| 10.98 | 132712 | 61 | C4 | 4734 | commelinids |
| 10.81 | 130730 | 41 | O | 38820 | Poales |
| 10.78 | 130370 | 947 | O1 | 4479 | Poaceae |
| 10.52 | 127140 | 0 | O2 | 147370 | PACMAD clade |
| 10.52 | 127140 | 540 | O3 | 147369 | Panicoideae |
| 10.42 | 126005 | 0 | O4 | 1648033 | Andropogonodae |
| 10.42 | 126005 | 772 | O5 | 147429 | Andropogoneae |
| 10.31 | 124596 | 0 | O6 | 1648029 | Tripsacinae |
| 10.31 | 124596 | 0 | G | 4575 | Zea |
| 10.31 | 124596 | 124596 | S | 4577 | Zea mays |

**Mixture 2**

| Percentage of reads within taxon | Number of reads within taxon | Number of reads identified as taxon | Taxonomic rank | NCBI taxon ID | Taxon |
| --- | --- | --- | --- | --- | --- |
| 37.15 | 401774 | 401774 | U | 0 | unclassified |
| 62.85 | 679753 | 0 | R | 1 | root |
| 62.85 | 679753 | 0 | R1 | 131567 | cellular organisms |
| 62.85 | 679753 | 14 | D | 2759 | Eukaryota |
| 62.85 | 679738 | 20 | K | 33090 | Viridiplantae |
| 62.85 | 679709 | 0 | P | 35493 | Streptophyta |
| 62.85 | 679709 | 0 | P1 | 131221 | Streptophytina |
| 62.85 | 679709 | 124 | P2 | 3193 | Embryophyta |
| 62.81 | 679260 | 0 | P3 | 58023 | Tracheophyta |
| 62.81 | 679260 | 0 | P4 | 78536 | Euphyllophyta |
| 62.81 | 679260 | 0 | P5 | 58024 | Spermatophyta |
| 62.81 | 679260 | 304 | C | 3398 | Magnoliopsida |
| 62.76 | 678719 | 5363 | C1 | 1437183 | Mesangiospermae |
| 33.36 | 360817 | 0 | C2 | 71240 | eudicotyledons |
| 33.36 | 360817 | 0 | C3 | 91827 | Gunneridae |
| 33.36 | 360817 | 4736 | C4 | 1437201 | Pentapetalae |
| 19.9 | 215269 | 671 | C5 | 71274 | asterids |
| 19.03 | 205829 | 79 | C6 | 91882 | campanulids |
| 18.97 | 205150 | 0 | O | 4209 | Asterales |
| 18.97 | 205150 | 561 | O1 | 4210 | Asteraceae |
| 18.63 | 201495 | 376 | O2 | 102804 | Asteroideae |
| 17.77 | 192230 | 0 | O3 | 102810 | Anthemideae |
| 17.77 | 192230 | 0 | O4 | 886714 | Artemisiinae |
| 17.77 | 192230 | 0 | G | 4219 | Artemisia |
| 17.77 | 192230 | 192230 | S | 55611 | Artemisia tridentata |
| 9 | 97286 | 809 | C5 | 71275 | rosids |
| 8.34 | 90209 | 2570 | C6 | 91835 | fabids |
| 4.64 | 50200 | 157 | O | 3744 | Rosales |
| 4.41 | 47714 | 0 | O1 | 3487 | Moraceae |
| 4.41 | 47714 | 0 | G | 66379 | Broussonetia |
| 4.41 | 47714 | 47714 | S | 172644 | Broussonetia papyrifera |
| 2.31 | 25009 | 27 | O | 3646 | Malpighiales |
| 2.27 | 24541 | 0 | O1 | 3688 | Salicaceae |
| 2.27 | 24541 | 0 | O2 | 238069 | Saliceae |
| 2.27 | 24541 | 1888 | G | 3689 | Populus |
| 1.62 | 17480 | 17480 | S | 3693 | Populus tremuloides |
| 4.02 | 43526 | 0 | O | 3524 | Caryophyllales |
| 4.02 | 43526 | 57 | O1 | 1804623 | Chenopodiaceae |
| 3.92 | 42407 | 0 | O2 | 1308824 | Camphorosmoideae |
| 3.92 | 42407 | 0 | O3 | 1308839 | Camphorosmeae |
| 3.92 | 42407 | 0 | G | 83153 | Bassia |
| 3.92 | 42407 | 42407 | S | 83154 | Bassia scoparia |
| 28.77 | 311209 | 0 | C2 | 4447 | Liliopsida |
| 28.77 | 311209 | 55 | C3 | 1437197 | Petrosaviidae |
| 28.7 | 310359 | 61 | C4 | 4734 | commelinids |
| 28.57 | 308980 | 44 | O | 38820 | Poales |
| 28.54 | 308660 | 1190 | O1 | 4479 | Poaceae |
| 22.26 | 240737 | 169 | O2 | 359160 | BOP clade |
| 22.14 | 239435 | 473 | O3 | 147368 | Pooideae |
| 21.81 | 235896 | 0 | O4 | 1648037 | Poodae |
| 21.81 | 235896 | 0 | O5 | 147387 | Poeae |
| 21.81 | 235896 | 0 | O6 | 1652081 | Poeae Chloroplast Group 2 (Poeae type) |
| 21.81 | 235896 | 0 | O7 | 640628 | Poinae |
| 21.81 | 235896 | 0 | G | 4544 | Poa |
| 21.81 | 235896 | 235896 | S | 4545 | Poa pratensis |
| 6.17 | 66733 | 0 | O2 | 147370 | PACMAD clade |
| 6.17 | 66733 | 480 | O3 | 147369 | Panicoideae |
| 5.97 | 64599 | 0 | O4 | 1648033 | Andropogonodae |
| 5.97 | 64599 | 426 | O5 | 147429 | Andropogoneae |
| 5.85 | 63242 | 0 | O6 | 1648029 | Tripsacinae |
| 5.85 | 63242 | 0 | G | 4575 | Zea |
| 5.85 | 63242 | 63242 | S | 4577 | Zea mays |

**Mixture 3**

| Percentage of reads within taxon | Number of reads within taxon | Number of reads identified as taxon | Taxonomic rank | NCBI taxon ID | Taxon |
| --- | --- | --- | --- | --- | --- |
| 25.42 | 218072 | 218072 | U | 0 | unclassified |
| 74.58 | 639808 | 0 | R | 1 | root |
| 74.58 | 639808 | 0 | R1 | 131567 | cellular organisms |
| 74.58 | 639808 | 5 | D | 2759 | Eukaryota |
| 74.58 | 639802 | 13 | K | 33090 | Viridiplantae |
| 74.58 | 639783 | 0 | P | 35493 | Streptophyta |
| 74.58 | 639783 | 0 | P1 | 131221 | Streptophytina |
| 74.58 | 639783 | 71 | P2 | 3193 | Embryophyta |
| 74.53 | 639420 | 0 | P3 | 58023 | Tracheophyta |
| 74.53 | 639420 | 0 | P4 | 78536 | Euphyllophyta |
| 74.53 | 639420 | 0 | P5 | 58024 | Spermatophyta |
| 74.53 | 639420 | 166 | C | 3398 | Magnoliopsida |
| 74.49 | 639028 | 3559 | C1 | 1437183 | Mesangiospermae |
| 44.36 | 380566 | 0 | C2 | 71240 | eudicotyledons |
| 44.36 | 380566 | 0 | C3 | 91827 | Gunneridae |
| 44.36 | 380566 | 4262 | C4 | 1437201 | Pentapetalae |
| 23.95 | 205484 | 661 | C5 | 71274 | asterids |
| 23 | 197279 | 89 | C6 | 91882 | campanulids |
| 22.93 | 196732 | 0 | O | 4209 | Asterales |
| 22.93 | 196732 | 477 | O1 | 4210 | Asteraceae |
| 22.52 | 193218 | 335 | O2 | 102804 | Asteroideae |
| 21.38 | 183408 | 0 | O3 | 102810 | Anthemideae |
| 21.38 | 183408 | 0 | O4 | 886714 | Artemisiinae |
| 21.38 | 183408 | 0 | G | 4219 | Artemisia |
| 21.38 | 183408 | 183408 | S | 55611 | Artemisia tridentata |
| 1.1 | 9475 | 0 | O3 | 911341 | Heliantheae alliance |
| 1.1 | 9475 | 65 | O4 | 102814 | Heliantheae |
| 1.07 | 9147 | 0 | G | 4231 | Helianthus |
| 1.07 | 9147 | 9147 | S | 4232 | Helianthus annuus |
| 16.93 | 145242 | 795 | C5 | 71275 | rosids |
| 16.21 | 139060 | 2255 | C6 | 91835 | fabids |
| 10.14 | 86985 | 128 | O | 3646 | Malpighiales |
| 10.07 | 86424 | 0 | O1 | 3688 | Salicaceae |
| 10.07 | 86424 | 0 | O2 | 238069 | Saliceae |
| 10.07 | 86424 | 15921 | G | 3689 | Populus |
| 7.51 | 64424 | 64424 | S | 3693 | Populus tremuloides |
| 3.39 | 29055 | 97 | O | 3744 | Rosales |
| 3.12 | 26790 | 0 | O1 | 3487 | Moraceae |
| 3.12 | 26790 | 0 | G | 66379 | Broussonetia |
| 3.12 | 26790 | 26790 | S | 172644 | Broussonetia papyrifera |
| 1.49 | 12771 | 28 | O | 3502 | Fagales |
| 1.4 | 11970 | 0 | O1 | 16714 | Juglandaceae |
| 1.4 | 11970 | 0 | G | 13402 | Carya |
| 1.4 | 11970 | 11970 | S | 32201 | Carya illinoinensis |
| 2.98 | 25578 | 0 | O | 3524 | Caryophyllales |
| 2.98 | 25578 | 33 | O1 | 1804623 | Chenopodiaceae |
| 2.87 | 24651 | 0 | O2 | 1308824 | Camphorosmoideae |
| 2.87 | 24651 | 0 | O3 | 1308839 | Camphorosmeae |
| 2.87 | 24651 | 0 | G | 83153 | Bassia |
| 2.87 | 24651 | 24651 | S | 83154 | Bassia scoparia |
| 29.58 | 253728 | 0 | C2 | 4447 | Liliopsida |
| 29.58 | 253728 | 33 | C3 | 1437197 | Petrosaviidae |
| 29.49 | 252953 | 57 | C4 | 4734 | commelinids |
| 29.34 | 251718 | 31 | O | 38820 | Poales |
| 29.31 | 251479 | 697 | O1 | 4479 | Poaceae |
| 25.4 | 217872 | 127 | O2 | 359160 | BOP clade |
| 25.26 | 216681 | 368 | O3 | 147368 | Pooideae |
| 24.89 | 213496 | 0 | O4 | 1648037 | Poodae |
| 24.89 | 213496 | 0 | O5 | 147387 | Poeae |
| 24.89 | 213496 | 0 | O6 | 1652081 | Poeae Chloroplast Group 2 (Poeae type) |
| 24.89 | 213496 | 0 | O7 | 640628 | Poinae |
| 24.89 | 213496 | 0 | G | 4544 | Poa |
| 24.89 | 213496 | 213496 | S | 4545 | Poa pratensis |
| 3.84 | 32910 | 0 | O2 | 147370 | PACMAD clade |
| 3.84 | 32910 | 231 | O3 | 147369 | Panicoideae |
| 3.63 | 31180 | 0 | O4 | 1648033 | Andropogonodae |
| 3.63 | 31180 | 172 | O5 | 147429 | Andropogoneae |
| 3.53 | 30249 | 0 | O6 | 1648029 | Tripsacinae |
| 3.53 | 30249 | 0 | G | 4575 | Zea |
| 3.53 | 30249 | 30249 | S | 4577 | Zea mays |

**Mixture 4**

| Percentage of reads within taxon | Number of reads within taxon | Number of reads identified as taxon | Taxonomic rank | NCBI taxon ID | Taxon |
| --- | --- | --- | --- | --- | --- |
| 23.94 | 214901 | 214901 | U | 0 | unclassified |
| 76.06 | 682921 | 0 | R | 1 | root |
| 76.06 | 682921 | 0 | R1 | 131567 | cellular organisms |
| 76.06 | 682921 | 2 | D | 2759 | Eukaryota |
| 76.06 | 682919 | 9 | K | 33090 | Viridiplantae |
| 76.06 | 682901 | 0 | P | 35493 | Streptophyta |
| 76.06 | 682901 | 0 | P1 | 131221 | Streptophytina |
| 76.06 | 682901 | 75 | P2 | 3193 | Embryophyta |
| 76.03 | 682576 | 0 | P3 | 58023 | Tracheophyta |
| 76.03 | 682576 | 0 | P4 | 78536 | Euphyllophyta |
| 76.03 | 682576 | 0 | P5 | 58024 | Spermatophyta |
| 76.03 | 682576 | 202 | C | 3398 | Magnoliopsida |
| 75.98 | 682143 | 3652 | C1 | 1437183 | Mesangiospermae |
| 49.52 | 444605 | 0 | C2 | 71240 | eudicotyledons |
| 49.52 | 444605 | 0 | C3 | 91827 | Gunneridae |
| 49.52 | 444605 | 4886 | C4 | 1437201 | Pentapetalae |
| 25.74 | 231108 | 697 | C5 | 71274 | asterids |
| 24.82 | 222814 | 112 | C6 | 91882 | campanulids |
| 24.75 | 222221 | 0 | O | 4209 | Asterales |
| 24.75 | 222221 | 590 | O1 | 4210 | Asteraceae |
| 24.33 | 218438 | 393 | O2 | 102804 | Asteroideae |
| 18.13 | 162741 | 0 | O3 | 102810 | Anthemideae |
| 18.13 | 162741 | 0 | O4 | 886714 | Artemisiinae |
| 18.13 | 162741 | 0 | G | 4219 | Artemisia |
| 18.13 | 162741 | 162741 | S | 55611 | Artemisia tridentata |
| 6.16 | 55304 | 0 | O3 | 911341 | Heliantheae alliance |
| 6.16 | 55304 | 384 | O4 | 102814 | Heliantheae |
| 3.69 | 33104 | 0 | G | 4211 | Ambrosia |
| 3.69 | 33104 | 33104 | S | 4212 | Ambrosia artemisiifolia |
| 2.43 | 21816 | 0 | G | 4231 | Helianthus |
| 2.43 | 21816 | 21816 | S | 4232 | Helianthus annuus |
| 20.49 | 184001 | 804 | C5 | 71275 | rosids |
| 19.8 | 177742 | 2658 | C6 | 91835 | fabids |
| 13.68 | 122860 | 140 | O | 3646 | Malpighiales |
| 13.62 | 122282 | 0 | O1 | 3688 | Salicaceae |
| 13.62 | 122282 | 0 | O2 | 238069 | Saliceae |
| 13.62 | 122282 | 26121 | G | 3689 | Populus |
| 7.33 | 65799 | 65799 | S | 3693 | Populus tremuloides |
| 3.28 | 29423 | 29423 | S | 3696 | Populus deltoides |
| 3.41 | 30651 | 99 | O | 3744 | Rosales |
| 3.16 | 28351 | 0 | O1 | 3487 | Moraceae |
| 3.16 | 28351 | 0 | G | 66379 | Broussonetia |
| 3.16 | 28351 | 28351 | S | 172644 | Broussonetia papyrifera |
| 1.54 | 13797 | 26 | O | 3502 | Fagales |
| 1.45 | 13016 | 0 | O1 | 16714 | Juglandaceae |
| 1.45 | 13016 | 0 | G | 13402 | Carya |
| 1.45 | 13016 | 13016 | S | 32201 | Carya illinoinensis |
| 2.74 | 24610 | 0 | O | 3524 | Caryophyllales |
| 2.74 | 24610 | 34 | O1 | 1804623 | Chenopodiaceae |
| 2.64 | 23666 | 0 | O2 | 1308824 | Camphorosmoideae |
| 2.64 | 23666 | 0 | O3 | 1308839 | Camphorosmeae |
| 2.64 | 23666 | 0 | G | 83153 | Bassia |
| 2.64 | 23666 | 23666 | S | 83154 | Bassia scoparia |
| 25.93 | 232767 | 0 | C2 | 4447 | Liliopsida |
| 25.93 | 232767 | 34 | C3 | 1437197 | Petrosaviidae |
| 25.84 | 232033 | 44 | C4 | 4734 | commelinids |
| 25.72 | 230895 | 20 | O | 38820 | Poales |
| 25.69 | 230648 | 678 | O1 | 4479 | Poaceae |
| 22.14 | 198811 | 120 | O2 | 359160 | BOP clade |
| 22.02 | 197737 | 378 | O3 | 147368 | Pooideae |
| 21.7 | 194788 | 0 | O4 | 1648037 | Poodae |
| 21.7 | 194788 | 0 | O5 | 147387 | Poeae |
| 21.7 | 194788 | 0 | O6 | 1652081 | Poeae Chloroplast Group 2 (Poeae type) |
| 21.7 | 194788 | 0 | O7 | 640628 | Poinae |
| 21.7 | 194788 | 0 | G | 4544 | Poa |
| 21.7 | 194788 | 194788 | S | 4545 | Poa pratensis |
| 3.47 | 31159 | 0 | O2 | 147370 | PACMAD clade |
| 3.47 | 31159 | 220 | O3 | 147369 | Panicoideae |
| 3.31 | 29700 | 0 | O4 | 1648033 | Andropogonodae |
| 3.31 | 29700 | 167 | O5 | 147429 | Andropogoneae |
| 3.2 | 28751 | 0 | O6 | 1648029 | Tripsacinae |
| 3.2 | 28751 | 0 | G | 4575 | Zea |
| 3.2 | 28751 | 28751 | S | 4577 | Zea mays |

**Mixture 5**

| Percentage of reads within taxon | Number of reads within taxon | Number of reads identified as taxon | Taxonomic rank | NCBI taxon ID | Taxon |
| --- | --- | --- | --- | --- | --- |
| 0.6 | 4498 | 4498 | U | 0 | unclassified |
| 99.4 | 744876 | 0 | R | 1 | root |
| 99.4 | 744876 | 0 | R1 | 131567 | cellular organisms |
| 99.4 | 744876 | 17 | D | 2759 | Eukaryota |
| 99.4 | 744858 | 12 | K | 33090 | Viridiplantae |
| 99.4 | 744844 | 0 | P | 35493 | Streptophyta |
| 99.4 | 744844 | 0 | P1 | 131221 | Streptophytina |
| 99.4 | 744844 | 54 | P2 | 3193 | Embryophyta |
| 99.39 | 744789 | 0 | P3 | 58023 | Tracheophyta |
| 99.39 | 744789 | 0 | P4 | 78536 | Euphyllophyta |
| 99.39 | 744789 | 0 | P5 | 58024 | Spermatophyta |
| 99.39 | 744789 | 537 | C | 3398 | Magnoliopsida |
| 99.32 | 744250 | 3217 | C1 | 1437183 | Mesangiospermae |
| 98.87 | 740874 | 0 | C2 | 71240 | eudicotyledons |
| 98.87 | 740874 | 0 | C3 | 91827 | Gunneridae |
| 98.87 | 740874 | 3594 | C4 | 1437201 | Pentapetalae |
| 98.36 | 737096 | 921 | C5 | 71275 | rosids |
| 98.23 | 736091 | 3967 | C6 | 91835 | fabids |
| 97.65 | 731770 | 1044 | O | 3646 | Malpighiales |
| 97.51 | 730702 | 0 | O1 | 3688 | Salicaceae |
| 97.51 | 730702 | 0 | O2 | 238069 | Saliceae |
| 97.51 | 730702 | 167688 | G | 3689 | Populus |
| 52.06 | 390115 | 390115 | S | 3693 | Populus tremuloides |
| 22.38 | 167702 | 167702 | S | 3696 | Populus deltoides |

**Mixture 6**

| Percentage of reads within taxon | Number of reads within taxon | Number of reads identified as taxon | Taxonomic rank | NCBI taxon ID | Taxon |
| --- | --- | --- | --- | --- | --- |
| 23.89 | 165024 | 165024 | U | 0 | unclassified |
| 76.11 | 525819 | 0 | R | 1 | root |
| 76.11 | 525819 | 0 | R1 | 131567 | cellular organisms |
| 76.11 | 525819 | 3 | D | 2759 | Eukaryota |
| 76.11 | 525815 | 6 | K | 33090 | Viridiplantae |
| 76.11 | 525803 | 0 | P | 35493 | Streptophyta |
| 76.11 | 525803 | 0 | P1 | 131221 | Streptophytina |
| 76.11 | 525803 | 38 | P2 | 3193 | Embryophyta |
| 76.08 | 525598 | 0 | P3 | 58023 | Tracheophyta |
| 76.08 | 525598 | 0 | P4 | 78536 | Euphyllophyta |
| 76.08 | 525598 | 0 | P5 | 58024 | Spermatophyta |
| 76.08 | 525598 | 59 | C | 3398 | Magnoliopsida |
| 76.05 | 525412 | 3639 | C1 | 1437183 | Mesangiospermae |
| 70.56 | 487448 | 0 | C2 | 4447 | Liliopsida |
| 70.56 | 487448 | 23 | C3 | 1437197 | Petrosaviidae |
| 70.52 | 487178 | 60 | C4 | 4734 | commelinids |
| 70.44 | 486616 | 42 | O | 38820 | Poales |
| 70.41 | 486437 | 1368 | O1 | 4479 | Poaceae |
| 59.52 | 411196 | 245 | O2 | 359160 | BOP clade |
| 59.26 | 409376 | 768 | O3 | 147368 | Pooideae |
| 58.36 | 403163 | 0 | O4 | 1648037 | Poodae |
| 58.36 | 403163 | 0 | O5 | 147387 | Poeae |
| 58.36 | 403163 | 0 | O6 | 1652081 | Poeae Chloroplast Group 2 (Poeae type) |
| 58.36 | 403163 | 0 | O7 | 640628 | Poinae |
| 58.36 | 403163 | 0 | G | 4544 | Poa |
| 58.36 | 403163 | 403163 | S | 4545 | Poa pratensis |
| 10.69 | 73873 | 0 | O2 | 147370 | PACMAD clade |
| 10.69 | 73873 | 448 | O3 | 147369 | Panicoideae |
| 10.29 | 71095 | 0 | O4 | 1648033 | Andropogonodae |
| 10.29 | 71095 | 360 | O5 | 147429 | Andropogoneae |
| 10.08 | 69638 | 0 | O6 | 1648029 | Tripsacinae |
| 10.08 | 69638 | 0 | G | 4575 | Zea |
| 10.08 | 69638 | 69638 | S | 4577 | Zea mays |
| 4.88 | 33709 | 0 | C2 | 71240 | eudicotyledons |
| 4.88 | 33709 | 0 | C3 | 91827 | Gunneridae |
| 4.88 | 33709 | 734 | C4 | 1437201 | Pentapetalae |
| 4.01 | 27686 | 286 | C5 | 71275 | rosids |
| 3.59 | 24769 | 761 | C6 | 91835 | fabids |
| 2.5 | 17240 | 9 | O | 3646 | Malpighiales |
| 2.47 | 17069 | 0 | O1 | 3688 | Salicaceae |
| 2.47 | 17069 | 0 | O2 | 238069 | Saliceae |
| 2.47 | 17069 | 664 | G | 3689 | Populus |
| 2.07 | 14319 | 14319 | S | 3693 | Populus tremuloides |

**Mixture 7**

| Percentage of reads within taxon | Number of reads within taxon | Number of reads identified as taxon | Taxonomic rank | NCBI taxon ID | Taxon |
| --- | --- | --- | --- | --- | --- |
| 34.57 | 307198 | 307198 | U | 0 | unclassified |
| 65.43 | 581375 | 0 | R | 1 | root |
| 65.43 | 581375 | 0 | R1 | 131567 | cellular organisms |
| 65.43 | 581375 | 3 | D | 2759 | Eukaryota |
| 65.43 | 581370 | 6 | K | 33090 | Viridiplantae |
| 65.43 | 581357 | 0 | P | 35493 | Streptophyta |
| 65.43 | 581357 | 0 | P1 | 131221 | Streptophytina |
| 65.43 | 581357 | 94 | P2 | 3193 | Embryophyta |
| 65.36 | 580806 | 0 | P3 | 58023 | Tracheophyta |
| 65.36 | 580806 | 0 | P4 | 78536 | Euphyllophyta |
| 65.36 | 580806 | 0 | P5 | 58024 | Spermatophyta |
| 65.36 | 580806 | 183 | C | 3398 | Magnoliopsida |
| 65.3 | 580267 | 3062 | C1 | 1437183 | Mesangiospermae |
| 63.86 | 567432 | 0 | C2 | 71240 | eudicotyledons |
| 63.86 | 567432 | 0 | C3 | 91827 | Gunneridae |
| 63.86 | 567432 | 7796 | C4 | 1437201 | Pentapetalae |
| 50.22 | 446242 | 1397 | C5 | 71274 | asterids |
| 48.56 | 431487 | 181 | C6 | 91882 | campanulids |
| 48.43 | 430378 | 0 | O | 4209 | Asterales |
| 48.43 | 430378 | 1093 | O1 | 4210 | Asteraceae |
| 47.62 | 423166 | 754 | O2 | 102804 | Asteroideae |
| 45.4 | 403448 | 0 | O3 | 102810 | Anthemideae |
| 45.4 | 403448 | 0 | O4 | 886714 | Artemisiinae |
| 45.4 | 403448 | 0 | G | 4219 | Artemisia |
| 45.4 | 403448 | 403448 | S | 55611 | Artemisia tridentata |
| 2.13 | 18964 | 0 | O3 | 911341 | Heliantheae alliance |
| 2.13 | 18964 | 152 | O4 | 102814 | Heliantheae |
| 2.05 | 18192 | 0 | G | 4231 | Helianthus |
| 2.05 | 18192 | 18192 | S | 4232 | Helianthus annuus |
| 1.5 | 13358 | 129 | C6 | 91888 | lamiids |
| 1.16 | 10268 | 33 | O | 4069 | Solanales |
| 1.07 | 9519 | 171 | O1 | 4070 | Solanaceae |
| 12.44 | 110501 | 1200 | C5 | 71275 | rosids |
| 11.26 | 100053 | 3299 | C6 | 91835 | fabids |
| 6.23 | 55314 | 141 | O | 3744 | Rosales |
| 5.79 | 51492 | 0 | O1 | 3487 | Moraceae |
| 5.79 | 51492 | 0 | G | 66379 | Broussonetia |
| 5.79 | 51492 | 51492 | S | 172644 | Broussonetia papyrifera |
| 2.74 | 24377 | 29 | O | 3646 | Malpighiales |
| 2.66 | 23667 | 0 | O1 | 3688 | Salicaceae |
| 2.66 | 23667 | 0 | O2 | 238069 | Saliceae |
| 2.66 | 23667 | 2459 | G | 3689 | Populus |
| 1.73 | 15363 | 15363 | S | 3693 | Populus tremuloides |
| 1.52 | 13478 | 0 | O | 72025 | Fabales |
| 1.52 | 13478 | 0 | O1 | 3803 | Fabaceae |
| 1.52 | 13478 | 0 | O2 | 3814 | Papilionoideae |
| 1.52 | 13478 | 319 | O3 | 2231393 | 50 kb inversion clade |

**Mixture 8**

| Percentage of reads within taxon | Number of reads within taxon | Number of reads identified as taxon | Taxonomic rank | NCBI taxon ID | Taxon |
| --- | --- | --- | --- | --- | --- |
| 23.54 | 167327 | 167327 | U | 0 | unclassified |
| 76.46 | 543432 | 0 | R | 1 | root |
| 76.46 | 543432 | 0 | R1 | 131567 | cellular organisms |
| 76.46 | 543432 | 3 | D | 2759 | Eukaryota |
| 76.46 | 543429 | 4 | K | 33090 | Viridiplantae |
| 76.46 | 543416 | 0 | P | 35493 | Streptophyta |
| 76.46 | 543416 | 0 | P1 | 131221 | Streptophytina |
| 76.46 | 543416 | 38 | P2 | 3193 | Embryophyta |
| 76.43 | 543199 | 0 | P3 | 58023 | Tracheophyta |
| 76.43 | 543199 | 0 | P4 | 78536 | Euphyllophyta |
| 76.43 | 543199 | 0 | P5 | 58024 | Spermatophyta |
| 76.43 | 543199 | 85 | C | 3398 | Magnoliopsida |
| 76.39 | 542975 | 3932 | C1 | 1437183 | Mesangiospermae |
| 63.49 | 451248 | 0 | C2 | 4447 | Liliopsida |
| 63.49 | 451248 | 39 | C3 | 1437197 | Petrosaviidae |
| 63.44 | 450881 | 69 | C4 | 4734 | commelinids |
| 63.34 | 450216 | 28 | O | 38820 | Poales |
| 63.32 | 450029 | 1051 | O1 | 4479 | Poaceae |
| 62.41 | 443573 | 289 | O2 | 359160 | BOP clade |
| 62.15 | 441704 | 862 | O3 | 147368 | Pooideae |
| 61.19 | 434903 | 0 | O4 | 1648037 | Poodae |
| 61.19 | 434903 | 0 | O5 | 147387 | Poeae |
| 61.19 | 434903 | 0 | O6 | 1652081 | Poeae Chloroplast Group 2 (Poeae type) |
| 61.19 | 434903 | 0 | O7 | 640628 | Poinae |
| 61.19 | 434903 | 0 | G | 4544 | Poa |
| 61.19 | 434903 | 434903 | S | 4545 | Poa pratensis |
| 12.26 | 87112 | 0 | C2 | 71240 | eudicotyledons |
| 12.26 | 87112 | 0 | C3 | 91827 | Gunneridae |
| 12.26 | 87112 | 1029 | C4 | 1437201 | Pentapetalae |
| 11.29 | 80278 | 383 | C5 | 71275 | rosids |
| 10.83 | 76943 | 1427 | C6 | 91835 | fabids |
| 7.3 | 51892 | 111 | O | 3744 | Rosales |
| 7.13 | 50643 | 0 | O1 | 3487 | Moraceae |
| 7.13 | 50643 | 0 | G | 66379 | Broussonetia |
| 7.13 | 50643 | 50643 | S | 172644 | Broussonetia papyrifera |
| 2.67 | 18947 | 8 | O | 3646 | Malpighiales |
| 2.63 | 18726 | 0 | O1 | 3688 | Salicaceae |
| 2.63 | 18726 | 0 | O2 | 238069 | Saliceae |
| 2.63 | 18726 | 827 | G | 3689 | Populus |
| 2.2 | 15667 | 15667 | S | 3693 | Populus tremuloides |

**Mixture 9**

| Percentage of reads within taxon | Number of reads within taxon | Number of reads identified as taxon | Taxonomic rank | NCBI taxon ID | Taxon |
| --- | --- | --- | --- | --- | --- |
| 25.24 | 154904 | 154904 | U | 0 | unclassified |
| 74.76 | 458933 | 0 | R | 1 | root |
| 74.76 | 458933 | 0 | R1 | 131567 | cellular organisms |
| 74.76 | 458933 | 2 | D | 2759 | Eukaryota |
| 74.76 | 458931 | 4 | K | 33090 | Viridiplantae |
| 74.76 | 458923 | 0 | P | 35493 | Streptophyta |
| 74.76 | 458923 | 0 | P1 | 131221 | Streptophytina |
| 74.76 | 458923 | 36 | P2 | 3193 | Embryophyta |
| 74.73 | 458750 | 0 | P3 | 58023 | Tracheophyta |
| 74.73 | 458750 | 0 | P4 | 78536 | Euphyllophyta |
| 74.73 | 458750 | 0 | P5 | 58024 | Spermatophyta |
| 74.73 | 458750 | 68 | C | 3398 | Magnoliopsida |
| 74.7 | 458556 | 3288 | C1 | 1437183 | Mesangiospermae |
| 67.48 | 414204 | 0 | C2 | 4447 | Liliopsida |
| 67.48 | 414204 | 24 | C3 | 1437197 | Petrosaviidae |
| 67.43 | 413894 | 68 | C4 | 4734 | commelinids |
| 67.33 | 413317 | 35 | O | 38820 | Poales |
| 67.3 | 413130 | 974 | O1 | 4479 | Poaceae |
| 66.35 | 407270 | 248 | O2 | 359160 | BOP clade |
| 66.07 | 405568 | 790 | O3 | 147368 | Pooideae |
| 65.03 | 399205 | 0 | O4 | 1648037 | Poodae |
| 65.03 | 399205 | 0 | O5 | 147387 | Poeae |
| 65.03 | 399205 | 0 | O6 | 1652081 | Poeae Chloroplast Group 2 (Poeae type) |
| 65.03 | 399205 | 0 | O7 | 640628 | Poinae |
| 65.03 | 399205 | 0 | G | 4544 | Poa |
| 65.03 | 399205 | 399205 | S | 4545 | Poa pratensis |
| 6.59 | 40479 | 0 | C2 | 71240 | eudicotyledons |
| 6.59 | 40479 | 0 | C3 | 91827 | Gunneridae |
| 6.59 | 40479 | 732 | C4 | 1437201 | Pentapetalae |
| 5.66 | 34726 | 279 | C5 | 71275 | rosids |
| 5.2 | 31899 | 848 | C6 | 91835 | fabids |
| 2.71 | 16632 | 4 | O | 3646 | Malpighiales |
| 2.68 | 16470 | 0 | O1 | 3688 | Salicaceae |
| 2.68 | 16470 | 0 | O2 | 238069 | Saliceae |
| 2.68 | 16470 | 710 | G | 3689 | Populus |
| 2.24 | 13769 | 13769 | S | 3693 | Populus tremuloides |
| 1.69 | 10369 | 30 | O | 3744 | Rosales |
| 1.52 | 9301 | 0 | O1 | 3487 | Moraceae |
| 1.52 | 9301 | 0 | G | 66379 | Broussonetia |
| 1.52 | 9301 | 9301 | S | 172644 | Broussonetia papyrifera |

**Mixture 10**

| Percentage of reads within taxon | Number of reads within taxon | Number of reads identified as taxon | Taxonomic rank | NCBI taxon ID | Taxon |
| --- | --- | --- | --- | --- | --- |
| 25.99 | 157045 | 157045 | U | 0 | unclassified |
| 74.01 | 447215 | 0 | R | 1 | root |
| 74.01 | 447215 | 0 | R1 | 131567 | cellular organisms |
| 74.01 | 447215 | 0 | D | 2759 | Eukaryota |
| 74.01 | 447214 | 2 | K | 33090 | Viridiplantae |
| 74.01 | 447199 | 0 | P | 35493 | Streptophyta |
| 74.01 | 447199 | 0 | P1 | 131221 | Streptophytina |
| 74.01 | 447199 | 26 | P2 | 3193 | Embryophyta |
| 73.98 | 447026 | 0 | P3 | 58023 | Tracheophyta |
| 73.98 | 447026 | 0 | P4 | 78536 | Euphyllophyta |
| 73.98 | 447026 | 0 | P5 | 58024 | Spermatophyta |
| 73.98 | 447026 | 64 | C | 3398 | Magnoliopsida |
| 73.95 | 446822 | 3393 | C1 | 1437183 | Mesangiospermae |
| 67.17 | 405888 | 0 | C2 | 4447 | Liliopsida |
| 67.17 | 405888 | 32 | C3 | 1437197 | Petrosaviidae |
| 67.12 | 405568 | 48 | C4 | 4734 | commelinids |
| 67.02 | 405002 | 34 | O | 38820 | Poales |
| 67 | 404833 | 973 | O1 | 4479 | Poaceae |
| 66.02 | 398917 | 265 | O2 | 359160 | BOP clade |
| 65.74 | 397224 | 777 | O3 | 147368 | Pooideae |
| 64.72 | 391069 | 0 | O4 | 1648037 | Poodae |
| 64.72 | 391069 | 0 | O5 | 147387 | Poeae |
| 64.72 | 391069 | 0 | O6 | 1652081 | Poeae Chloroplast Group 2 (Poeae type) |
| 64.72 | 391069 | 0 | O7 | 640628 | Poinae |
| 64.72 | 391069 | 0 | G | 4544 | Poa |
| 64.72 | 391069 | 391069 | S | 4545 | Poa pratensis |
| 6.11 | 36938 | 0 | C2 | 71240 | eudicotyledons |
| 6.11 | 36938 | 0 | C3 | 91827 | Gunneridae |
| 6.11 | 36938 | 748 | C4 | 1437201 | Pentapetalae |
| 5.17 | 31257 | 256 | C5 | 71275 | rosids |
| 4.69 | 28343 | 766 | C6 | 91835 | fabids |
| 2.76 | 16705 | 6 | O | 3646 | Malpighiales |
| 2.73 | 16519 | 0 | O1 | 3688 | Salicaceae |
| 2.73 | 16519 | 0 | O2 | 238069 | Saliceae |
| 2.73 | 16519 | 664 | G | 3689 | Populus |
| 2.3 | 13874 | 13874 | S | 3693 | Populus tremuloides |
| 1.13 | 6824 | 18 | O | 3744 | Rosales |

**Mixture 11**

| Percentage of reads within taxon | Number of reads within taxon | Number of reads identified as taxon | Taxonomic rank | NCBI taxon ID | Taxon |
| --- | --- | --- | --- | --- | --- |
| 26.31 | 240436 | 240436 | U | 0 | unclassified |
| 73.69 | 673577 | 0 | R | 1 | root |
| 73.69 | 673577 | 0 | R1 | 131567 | cellular organisms |
| 73.69 | 673577 | 4 | D | 2759 | Eukaryota |
| 73.69 | 673571 | 2 | K | 33090 | Viridiplantae |
| 73.69 | 673554 | 0 | P | 35493 | Streptophyta |
| 73.69 | 673554 | 0 | P1 | 131221 | Streptophytina |
| 73.69 | 673554 | 62 | P2 | 3193 | Embryophyta |
| 73.66 | 673243 | 0 | P3 | 58023 | Tracheophyta |
| 73.66 | 673243 | 0 | P4 | 78536 | Euphyllophyta |
| 73.66 | 673243 | 0 | P5 | 58024 | Spermatophyta |
| 73.66 | 673243 | 78 | C | 3398 | Magnoliopsida |
| 73.63 | 672980 | 5113 | C1 | 1437183 | Mesangiospermae |
| 66.93 | 611721 | 0 | C2 | 4447 | Liliopsida |
| 66.93 | 611721 | 27 | C3 | 1437197 | Petrosaviidae |
| 66.88 | 611261 | 79 | C4 | 4734 | commelinids |
| 66.78 | 610349 | 31 | O | 38820 | Poales |
| 66.75 | 610117 | 1494 | O1 | 4479 | Poaceae |
| 65.8 | 601408 | 376 | O2 | 359160 | BOP clade |
| 65.52 | 598871 | 1180 | O3 | 147368 | Pooideae |
| 64.5 | 589553 | 0 | O4 | 1648037 | Poodae |
| 64.5 | 589553 | 0 | O5 | 147387 | Poeae |
| 64.5 | 589553 | 0 | O6 | 1652081 | Poeae Chloroplast Group 2 (Poeae type) |
| 64.5 | 589553 | 0 | O7 | 640628 | Poinae |
| 64.5 | 589553 | 0 | G | 4544 | Poa |
| 64.5 | 589553 | 589553 | S | 4545 | Poa pratensis |
| 6.04 | 55248 | 0 | C2 | 71240 | eudicotyledons |
| 6.04 | 55248 | 0 | C3 | 91827 | Gunneridae |
| 6.04 | 55248 | 1166 | C4 | 1437201 | Pentapetalae |
| 5.07 | 46377 | 408 | C5 | 71275 | rosids |
| 4.59 | 41967 | 1222 | C6 | 91835 | fabids |
| 2.78 | 25437 | 10 | O | 3646 | Malpighiales |
| 2.75 | 25141 | 0 | O1 | 3688 | Salicaceae |
| 2.75 | 25141 | 0 | O2 | 238069 | Saliceae |
| 2.75 | 25141 | 1059 | G | 3689 | Populus |
| 2.29 | 20962 | 20962 | S | 3693 | Populus tremuloides |

***Ambrosia artemisiifolia***

| Percentage of reads within taxon | Number of reads within taxon | Number of reads identified as taxon | Taxonomic rank | NCBI taxon ID | Taxon |
| --- | --- | --- | --- | --- | --- |
| 30.06 | 282171 | 282171 | U | 0 | unclassified |
| 69.94 | 656579 | 0 | R | 1 | root |
| 69.94 | 656579 | 0 | R1 | 131567 | cellular organisms |
| 69.94 | 656579 | 6 | D | 2759 | Eukaryota |
| 69.94 | 656573 | 7 | K | 33090 | Viridiplantae |
| 69.94 | 656555 | 0 | P | 35493 | Streptophyta |
| 69.94 | 656555 | 0 | P1 | 131221 | Streptophytina |
| 69.94 | 656555 | 158 | P2 | 3193 | Embryophyta |
| 69.87 | 655904 | 0 | P3 | 58023 | Tracheophyta |
| 69.87 | 655904 | 0 | P4 | 78536 | Euphyllophyta |
| 69.87 | 655904 | 0 | P5 | 58024 | Spermatophyta |
| 69.87 | 655904 | 170 | C | 3398 | Magnoliopsida |
| 69.82 | 655398 | 2741 | C1 | 1437183 | Mesangiospermae |
| 68.71 | 645053 | 0 | C2 | 71240 | eudicotyledons |
| 68.71 | 645053 | 0 | C3 | 91827 | Gunneridae |
| 68.71 | 645053 | 8996 | C4 | 1437201 | Pentapetalae |
| 60.02 | 563438 | 1191 | C5 | 71274 | asterids |
| 58.77 | 551719 | 292 | C6 | 91882 | campanulids |
| 58.65 | 550617 | 0 | O | 4209 | Asterales |
| 58.65 | 550617 | 1766 | O1 | 4210 | Asteraceae |
| 57.93 | 543810 | 904 | O2 | 102804 | Asteroideae |
| 57.51 | 539854 | 0 | O3 | 911341 | Heliantheae alliance |
| 57.51 | 539854 | 3649 | O4 | 102814 | Heliantheae |
| 40.04 | 375837 | 0 | G | 4211 | Ambrosia |
| 40.04 | 375837 | 375837 | S | 4212 | Ambrosia artemisiifolia |
| 17.08 | 160368 | 0 | G | 4231 | Helianthus |
| 17.08 | 160368 | 160368 | S | 4232 | Helianthus annuus |
| 1.12 | 10528 | 80 | C6 | 91888 | lamiids |
| 7.41 | 69604 | 895 | C5 | 71275 | rosids |
| 6.56 | 61548 | 3995 | C6 | 91835 | fabids |
| 3.99 | 37492 | 36 | O | 3646 | Malpighiales |
| 3.93 | 36926 | 0 | O1 | 3688 | Salicaceae |
| 3.93 | 36926 | 0 | O2 | 238069 | Saliceae |
| 3.93 | 36926 | 3934 | G | 3689 | Populus |
| 2.6 | 24369 | 24369 | S | 3693 | Populus tremuloides |
| 1.01 | 9525 | 0 | O | 72025 | Fabales |
| 1.01 | 9525 | 0 | O1 | 3803 | Fabaceae |
| 1.01 | 9525 | 0 | O2 | 3814 | Papilionoideae |
| 1.01 | 9525 | 148 | O3 | 2231393 | 50 kb inversion clade |

***Artemisia tridentata***

| Percentage of reads within taxon | Number of reads within taxon | Number of reads identified as taxon | Taxonomic rank | NCBI taxon ID | Taxon |
| --- | --- | --- | --- | --- | --- |
| 35.58 | 306338 | 306338 | U | 0 | unclassified |
| 64.42 | 554707 | 0 | R | 1 | root |
| 64.42 | 554707 | 0 | R1 | 131567 | cellular organisms |
| 64.42 | 554707 | 3 | D | 2759 | Eukaryota |
| 64.42 | 554701 | 7 | K | 33090 | Viridiplantae |
| 64.42 | 554686 | 0 | P | 35493 | Streptophyta |
| 64.42 | 554686 | 0 | P1 | 131221 | Streptophytina |
| 64.42 | 554686 | 86 | P2 | 3193 | Embryophyta |
| 64.36 | 554175 | 0 | P3 | 58023 | Tracheophyta |
| 64.36 | 554175 | 0 | P4 | 78536 | Euphyllophyta |
| 64.36 | 554175 | 0 | P5 | 58024 | Spermatophyta |
| 64.36 | 554175 | 139 | C | 3398 | Magnoliopsida |
| 64.31 | 553707 | 3006 | C1 | 1437183 | Mesangiospermae |
| 62.83 | 540984 | 0 | C2 | 71240 | eudicotyledons |
| 62.83 | 540984 | 0 | C3 | 91827 | Gunneridae |
| 62.83 | 540984 | 7681 | C4 | 1437201 | Pentapetalae |
| 54.72 | 471138 | 1533 | C5 | 71274 | asterids |
| 52.97 | 456103 | 220 | C6 | 91882 | campanulids |
| 52.83 | 454912 | 0 | O | 4209 | Asterales |
| 52.83 | 454912 | 1120 | O1 | 4210 | Asteraceae |
| 51.93 | 447116 | 778 | O2 | 102804 | Asteroideae |
| 49.42 | 425518 | 0 | O3 | 102810 | Anthemideae |
| 49.42 | 425518 | 0 | O4 | 886714 | Artemisiinae |
| 49.42 | 425518 | 0 | G | 4219 | Artemisia |
| 49.42 | 425518 | 425518 | S | 55611 | Artemisia tridentata |
| 2.42 | 20820 | 0 | O3 | 911341 | Heliantheae alliance |
| 2.42 | 20820 | 156 | O4 | 102814 | Heliantheae |
| 2.33 | 20103 | 0 | G | 4231 | Helianthus |
| 2.33 | 20103 | 20103 | S | 4232 | Helianthus annuus |
| 1.57 | 13502 | 139 | C6 | 91888 | lamiids |
| 1.2 | 10351 | 33 | O | 4069 | Solanales |
| 1.1 | 9490 | 201 | O1 | 4070 | Solanaceae |
| 6.89 | 59334 | 1123 | C5 | 71275 | rosids |
| 5.66 | 48701 | 2558 | C6 | 91835 | fabids |
| 2.6 | 22359 | 37 | O | 3646 | Malpighiales |
| 2.51 | 21621 | 0 | O1 | 3688 | Salicaceae |
| 2.51 | 21621 | 0 | O2 | 238069 | Saliceae |
| 2.51 | 21621 | 2188 | G | 3689 | Populus |
| 1.62 | 13960 | 13960 | S | 3693 | Populus tremuloides |
| 1.61 | 13830 | 0 | O | 72025 | Fabales |
| 1.61 | 13830 | 0 | O1 | 3803 | Fabaceae |
| 1.61 | 13830 | 0 | O2 | 3814 | Papilionoideae |
| 1.61 | 13830 | 347 | O3 | 2231393 | 50 kb inversion clade |
| 1.04 | 8994 | 159 | O4 | 2231382 | NPAAA clade |
| 1.03 | 8835 | 68 | C6 | 91836 | malvids |

***Broussonetia papyrifera***

| Percentage of reads within taxon | Number of reads within taxon | Number of reads identified as taxon | Taxonomic rank | NCBI taxon ID | Taxon |
| --- | --- | --- | --- | --- | --- |
| 28.57 | 2 | 2 | U | 0 | unclassified |
| 71.43 | 5 | 0 | R | 1 | root |
| 71.43 | 5 | 0 | R1 | 131567 | cellular organisms |
| 71.43 | 5 | 0 | D | 2759 | Eukaryota |
| 71.43 | 5 | 0 | K | 33090 | Viridiplantae |
| 71.43 | 5 | 0 | P | 35493 | Streptophyta |
| 71.43 | 5 | 0 | P1 | 131221 | Streptophytina |
| 71.43 | 5 | 0 | P2 | 3193 | Embryophyta |
| 71.43 | 5 | 0 | P3 | 58023 | Tracheophyta |
| 71.43 | 5 | 0 | P4 | 78536 | Euphyllophyta |
| 71.43 | 5 | 0 | P5 | 58024 | Spermatophyta |
| 71.43 | 5 | 0 | C | 3398 | Magnoliopsida |
| 71.43 | 5 | 0 | C1 | 1437183 | Mesangiospermae |
| 57.14 | 4 | 0 | C2 | 4447 | Liliopsida |
| 57.14 | 4 | 0 | C3 | 1437197 | Petrosaviidae |
| 57.14 | 4 | 0 | C4 | 4734 | commelinids |
| 57.14 | 4 | 0 | O | 38820 | Poales |
| 57.14 | 4 | 0 | O1 | 4479 | Poaceae |
| 28.57 | 2 | 0 | O2 | 147370 | PACMAD clade |
| 28.57 | 2 | 0 | O3 | 147369 | Panicoideae |
| 14.29 | 1 | 0 | O4 | 1648033 | Andropogonodae |
| 14.29 | 1 | 0 | O5 | 147429 | Andropogoneae |
| 14.29 | 1 | 0 | O6 | 1648029 | Tripsacinae |
| 14.29 | 1 | 0 | G | 4575 | Zea |
| 14.29 | 1 | 1 | S | 4577 | Zea mays |
| 14.29 | 1 | 0 | O4 | 1648036 | Panicodae |
| 14.29 | 1 | 0 | O5 | 147428 | Paniceae |
| 14.29 | 1 | 0 | O6 | 1293361 | Cenchrinae |
| 14.29 | 1 | 0 | G | 4554 | Setaria |
| 14.29 | 1 | 1 | S | 4555 | Setaria italica |
| 28.57 | 2 | 0 | O2 | 359160 | BOP clade |
| 28.57 | 2 | 0 | O3 | 147368 | Pooideae |
| 28.57 | 2 | 0 | O4 | 1648037 | Poodae |
| 28.57 | 2 | 0 | O5 | 147387 | Poeae |
| 28.57 | 2 | 0 | O6 | 1652081 | Poeae Chloroplast Group 2 (Poeae type) |
| 28.57 | 2 | 0 | O7 | 640628 | Poinae |
| 28.57 | 2 | 0 | G | 4544 | Poa |
| 28.57 | 2 | 2 | S | 4545 | Poa pratensis |
| 14.29 | 1 | 0 | C2 | 71240 | eudicotyledons |
| 14.29 | 1 | 0 | C3 | 91827 | Gunneridae |
| 14.29 | 1 | 0 | C4 | 1437201 | Pentapetalae |
| 14.29 | 1 | 0 | C5 | 71275 | rosids |
| 14.29 | 1 | 0 | C6 | 91835 | fabids |
| 14.29 | 1 | 0 | O | 3744 | Rosales |
| 14.29 | 1 | 0 | O1 | 3745 | Rosaceae |
| 14.29 | 1 | 0 | O2 | 171638 | Rosoideae |
| 14.29 | 1 | 0 | O3 | 721789 | Potentilleae |
| 14.29 | 1 | 0 | O4 | 1184124 | Fragariinae |
| 14.29 | 1 | 0 | G | 3746 | Fragaria |
| 14.29 | 1 | 0 | S | 57918 | Fragaria vesca |
| 14.29 | 1 | 1 | S1 | 101020 | Fragaria vesca subsp. vesca |

***Bassia scoparia***

| Percentage of reads within taxon | Number of reads within taxon | Number of reads identified as taxon | Taxonomic rank | NCBI taxon ID | Taxon |
| --- | --- | --- | --- | --- | --- |
| 3.2 | 27764 | 27764 | U | 0 | unclassified |
| 96.8 | 839951 | 0 | R | 1 | root |
| 96.8 | 839951 | 0 | R1 | 131567 | cellular organisms |
| 96.8 | 839951 | 8 | D | 2759 | Eukaryota |
| 96.8 | 839943 | 15 | K | 33090 | Viridiplantae |
| 96.8 | 839922 | 0 | P | 35493 | Streptophyta |
| 96.8 | 839922 | 0 | P1 | 131221 | Streptophytina |
| 96.8 | 839922 | 70 | P2 | 3193 | Embryophyta |
| 96.78 | 839814 | 0 | P3 | 58023 | Tracheophyta |
| 96.78 | 839814 | 0 | P4 | 78536 | Euphyllophyta |
| 96.78 | 839814 | 0 | P5 | 58024 | Spermatophyta |
| 96.78 | 839814 | 208 | C | 3398 | Magnoliopsida |
| 96.76 | 839568 | 1143 | C1 | 1437183 | Mesangiospermae |
| 96.49 | 837221 | 0 | C2 | 71240 | eudicotyledons |
| 96.49 | 837221 | 0 | C3 | 91827 | Gunneridae |
| 96.49 | 837221 | 3858 | C4 | 1437201 | Pentapetalae |
| 94.57 | 820594 | 0 | O | 3524 | Caryophyllales |
| 94.57 | 820594 | 576 | O1 | 1804623 | Chenopodiaceae |
| 93.65 | 812621 | 0 | O2 | 1308824 | Camphorosmoideae |
| 93.65 | 812621 | 0 | O3 | 1308839 | Camphorosmeae |
| 93.65 | 812621 | 0 | G | 83153 | Bassia |
| 93.65 | 812621 | 812621 | S | 83154 | Bassia scoparia |
| 1.22 | 10597 | 201 | C5 | 71275 | rosids |
| 1.07 | 9251 | 602 | C6 | 91835 | fabids |

***Carya illinoinensis***

| Percentage of reads within taxon | Number of reads within taxon | Number of reads identified as taxon | Taxonomic rank | NCBI taxon ID | Taxon |
| --- | --- | --- | --- | --- | --- |
| 4.77 | 64093 | 64093 | U | 0 | unclassified |
| 95.23 | 1279514 | 0 | R | 1 | root |
| 95.23 | 1279514 | 0 | R1 | 131567 | cellular organisms |
| 95.23 | 1279514 | 34 | D | 2759 | Eukaryota |
| 95.23 | 1279479 | 41 | K | 33090 | Viridiplantae |
| 95.22 | 1279428 | 0 | P | 35493 | Streptophyta |
| 95.22 | 1279428 | 0 | P1 | 131221 | Streptophytina |
| 95.22 | 1279428 | 216 | P2 | 3193 | Embryophyta |
| 95.2 | 1279172 | 0 | P3 | 58023 | Tracheophyta |
| 95.2 | 1279172 | 0 | P4 | 78536 | Euphyllophyta |
| 95.2 | 1279172 | 0 | P5 | 58024 | Spermatophyta |
| 95.2 | 1279172 | 1083 | C | 3398 | Magnoliopsida |
| 95.12 | 1278062 | 6428 | C1 | 1437183 | Mesangiospermae |
| 94.45 | 1268997 | 0 | C2 | 71240 | eudicotyledons |
| 94.45 | 1268997 | 0 | C3 | 91827 | Gunneridae |
| 94.45 | 1268997 | 4653 | C4 | 1437201 | Pentapetalae |
| 93.96 | 1262518 | 2071 | C5 | 71275 | rosids |
| 93.75 | 1259636 | 8666 | C6 | 91835 | fabids |
| 91.52 | 1229606 | 2061 | O | 3502 | Fagales |
| 91.24 | 1225924 | 0 | O1 | 16714 | Juglandaceae |
| 91.24 | 1225924 | 0 | G | 13402 | Carya |
| 91.24 | 1225924 | 1225924 | S | 32201 | Carya illinoinensis |
| 1.19 | 16019 | 14 | O | 3646 | Malpighiales |
| 1.19 | 15950 | 0 | O1 | 3688 | Salicaceae |
| 1.19 | 15950 | 0 | O2 | 238069 | Saliceae |
| 1.19 | 15950 | 3063 | G | 3689 | Populus |

***Populus deltoides***

| Percentage of reads within taxon | Number of reads within taxon | Number of reads identified as taxon | Taxonomic rank | NCBI taxon ID | Taxon |
| --- | --- | --- | --- | --- | --- |
| 1.17 | 9194 | 9194 | U | 0 | unclassified |
| 98.83 | 773325 | 0 | R | 1 | root |
| 98.83 | 773325 | 0 | R1 | 131567 | cellular organisms |
| 98.83 | 773325 | 2 | D | 2759 | Eukaryota |
| 98.82 | 773322 | 8 | K | 33090 | Viridiplantae |
| 98.82 | 773312 | 0 | P | 35493 | Streptophyta |
| 98.82 | 773312 | 0 | P1 | 131221 | Streptophytina |
| 98.82 | 773312 | 59 | P2 | 3193 | Embryophyta |
| 98.82 | 773248 | 0 | P3 | 58023 | Tracheophyta |
| 98.82 | 773248 | 0 | P4 | 78536 | Euphyllophyta |
| 98.82 | 773248 | 0 | P5 | 58024 | Spermatophyta |
| 98.82 | 773248 | 509 | C | 3398 | Magnoliopsida |
| 98.75 | 772734 | 3469 | C1 | 1437183 | Mesangiospermae |
| 98.28 | 769045 | 0 | C2 | 71240 | eudicotyledons |
| 98.28 | 769045 | 0 | C3 | 91827 | Gunneridae |
| 98.28 | 769045 | 3818 | C4 | 1437201 | Pentapetalae |
| 97.76 | 764963 | 1165 | C5 | 71275 | rosids |
| 97.6 | 763721 | 4268 | C6 | 91835 | fabids |
| 96.97 | 758828 | 1101 | O | 3646 | Malpighiales |
| 96.83 | 757720 | 0 | O1 | 3688 | Salicaceae |
| 96.83 | 757720 | 0 | O2 | 238069 | Saliceae |
| 96.83 | 757720 | 202426 | G | 3689 | Populus |
| 62.42 | 488426 | 488426 | S | 3696 | Populus deltoides |
| 7.68 | 60101 | 60101 | S | 3693 | Populus tremuloides |

***Poa pratensis***

| Percentage of reads within taxon | Number of reads within taxon | Number of reads identified as taxon | Taxonomic rank | NCBI taxon ID | Taxon |
| --- | --- | --- | --- | --- | --- |
| 26.28 | 228193 | 228193 | U | 0 | unclassified |
| 73.72 | 640084 | 0 | R | 1 | root |
| 73.72 | 640084 | 0 | R1 | 131567 | cellular organisms |
| 73.72 | 640084 | 1 | D | 2759 | Eukaryota |
| 73.72 | 640083 | 6 | K | 33090 | Viridiplantae |
| 73.72 | 640065 | 0 | P | 35493 | Streptophyta |
| 73.72 | 640065 | 0 | P1 | 131221 | Streptophytina |
| 73.72 | 640065 | 53 | P2 | 3193 | Embryophyta |
| 73.68 | 639788 | 0 | P3 | 58023 | Tracheophyta |
| 73.68 | 639788 | 0 | P4 | 78536 | Euphyllophyta |
| 73.68 | 639788 | 0 | P5 | 58024 | Spermatophyta |
| 73.68 | 639788 | 78 | C | 3398 | Magnoliopsida |
| 73.65 | 639504 | 4921 | C1 | 1437183 | Mesangiospermae |
| 67.18 | 583298 | 0 | C2 | 4447 | Liliopsida |
| 67.18 | 583298 | 41 | C3 | 1437197 | Petrosaviidae |
| 67.13 | 582867 | 83 | C4 | 4734 | commelinids |
| 67.03 | 582013 | 28 | O | 38820 | Poales |
| 67.01 | 581800 | 1392 | O1 | 4479 | Poaceae |
| 66.07 | 573714 | 329 | O2 | 359160 | BOP clade |
| 65.82 | 571511 | 1110 | O3 | 147368 | Pooideae |
| 64.81 | 562692 | 0 | O4 | 1648037 | Poodae |
| 64.81 | 562692 | 0 | O5 | 147387 | Poeae |
| 64.81 | 562692 | 0 | O6 | 1652081 | Poeae Chloroplast Group 2 (Poeae type) |
| 64.81 | 562692 | 0 | O7 | 640628 | Poinae |
| 64.81 | 562692 | 0 | G | 4544 | Poa |
| 64.81 | 562692 | 562692 | S | 4545 | Poa pratensis |
| 5.8 | 50365 | 0 | C2 | 71240 | eudicotyledons |
| 5.8 | 50365 | 0 | C3 | 91827 | Gunneridae |
| 5.8 | 50365 | 1120 | C4 | 1437201 | Pentapetalae |
| 4.8 | 41634 | 383 | C5 | 71275 | rosids |
| 4.29 | 37208 | 1202 | C6 | 91835 | fabids |
| 3 | 26016 | 14 | O | 3646 | Malpighiales |
| 2.96 | 25706 | 0 | O1 | 3688 | Salicaceae |
| 2.96 | 25706 | 0 | O2 | 238069 | Saliceae |
| 2.96 | 25706 | 1127 | G | 3689 | Populus |
| 2.48 | 21504 | 21504 | S | 3693 | Populus tremuloides |

***Populus tremuloides***

| Percentage of reads within taxon | Number of reads within taxon | Number of reads identified as taxon | Taxonomic rank | NCBI taxon ID | Taxon |
| --- | --- | --- | --- | --- | --- |
| 0.25 | 2016 | 2016 | U | 0 | unclassified |
| 99.75 | 804651 | 0 | R | 1 | root |
| 99.75 | 804651 | 0 | R1 | 131567 | cellular organisms |
| 99.75 | 804651 | 3 | D | 2759 | Eukaryota |
| 99.75 | 804648 | 6 | K | 33090 | Viridiplantae |
| 99.75 | 804641 | 0 | P | 35493 | Streptophyta |
| 99.75 | 804641 | 0 | P1 | 131221 | Streptophytina |
| 99.75 | 804641 | 71 | P2 | 3193 | Embryophyta |
| 99.74 | 804568 | 0 | P3 | 58023 | Tracheophyta |
| 99.74 | 804568 | 0 | P4 | 78536 | Euphyllophyta |
| 99.74 | 804568 | 0 | P5 | 58024 | Spermatophyta |
| 99.74 | 804568 | 482 | C | 3398 | Magnoliopsida |
| 99.68 | 804086 | 2892 | C1 | 1437183 | Mesangiospermae |
| 99.3 | 801034 | 0 | C2 | 71240 | eudicotyledons |
| 99.3 | 801034 | 0 | C3 | 91827 | Gunneridae |
| 99.3 | 801034 | 3368 | C4 | 1437201 | Pentapetalae |
| 98.85 | 797429 | 1020 | C5 | 71275 | rosids |
| 98.71 | 796290 | 3681 | C6 | 91835 | fabids |
| 98.22 | 792334 | 1192 | O | 3646 | Malpighiales |
| 98.07 | 791109 | 0 | O1 | 3688 | Salicaceae |
| 98.07 | 791109 | 0 | O2 | 238069 | Saliceae |
| 98.07 | 791109 | 167108 | G | 3689 | Populus |
| 73.56 | 593385 | 593385 | S | 3693 | Populus tremuloides |
| 3.17 | 25595 | 25595 | S | 3696 | Populus deltoides |

***Zea mays***

| Percentage of reads within taxon | Number of reads within taxon | Number of reads identified as taxon | Taxonomic rank | NCBI taxon ID | Taxon |
| --- | --- | --- | --- | --- | --- |
| 2.61 | 18781 | 18781 | U | 0 | unclassified |
| 97.39 | 700507 | 0 | R | 1 | root |
| 97.39 | 700507 | 0 | R1 | 131567 | cellular organisms |
| 97.39 | 700507 | 12 | D | 2759 | Eukaryota |
| 97.39 | 700495 | 4 | K | 33090 | Viridiplantae |
| 97.39 | 700488 | 0 | P | 35493 | Streptophyta |
| 97.39 | 700488 | 0 | P1 | 131221 | Streptophytina |
| 97.39 | 700488 | 8 | P2 | 3193 | Embryophyta |
| 97.38 | 700469 | 0 | P3 | 58023 | Tracheophyta |
| 97.38 | 700469 | 0 | P4 | 78536 | Euphyllophyta |
| 97.38 | 700469 | 0 | P5 | 58024 | Spermatophyta |
| 97.38 | 700469 | 59 | C | 3398 | Magnoliopsida |
| 97.37 | 700395 | 2198 | C1 | 1437183 | Mesangiospermae |
| 96.41 | 693434 | 0 | C2 | 4447 | Liliopsida |
| 96.41 | 693434 | 29 | C3 | 1437197 | Petrosaviidae |
| 96.4 | 693385 | 42 | C4 | 4734 | commelinids |
| 96.39 | 693304 | 87 | O | 38820 | Poales |
| 96.37 | 693212 | 3501 | O1 | 4479 | Poaceae |
| 95.75 | 688708 | 0 | O2 | 147370 | PACMAD clade |
| 95.75 | 688708 | 1874 | O3 | 147369 | Panicoideae |
| 95.45 | 686545 | 0 | O4 | 1648033 | Andropogonodae |
| 95.45 | 686545 | 3055 | O5 | 147429 | Andropogoneae |
| 94.97 | 683114 | 0 | O6 | 1648029 | Tripsacinae |
| 94.97 | 683114 | 0 | G | 4575 | Zea |
| 94.97 | 683114 | 683114 | S | 4577 | Zea mays |

**Negative control 1**

| Percentage of reads within taxon | Number of reads within taxon | Number of reads identified as taxon | Taxonomic rank | NCBI taxon ID | Taxon |
| --- | --- | --- | --- | --- | --- |
| 54.35 | 181 | 181 | U | 0 | unclassified |
| 45.65 | 152 | 0 | R | 1 | root |
| 45.65 | 152 | 0 | R1 | 131567 | cellular organisms |
| 45.65 | 152 | 0 | D | 2759 | Eukaryota |
| 45.65 | 152 | 0 | K | 33090 | Viridiplantae |
| 45.65 | 152 | 0 | P | 35493 | Streptophyta |
| 45.65 | 152 | 0 | P1 | 131221 | Streptophytina |
| 45.65 | 152 | 0 | P2 | 3193 | Embryophyta |
| 45.65 | 152 | 0 | P3 | 58023 | Tracheophyta |
| 45.65 | 152 | 0 | P4 | 78536 | Euphyllophyta |
| 45.65 | 152 | 0 | P5 | 58024 | Spermatophyta |
| 45.65 | 152 | 0 | C | 3398 | Magnoliopsida |
| 45.65 | 152 | 5 | C1 | 1437183 | Mesangiospermae |
| 33.63 | 112 | 0 | C2 | 71240 | eudicotyledons |
| 33.63 | 112 | 0 | C3 | 91827 | Gunneridae |
| 33.63 | 112 | 2 | C4 | 1437201 | Pentapetalae |
| 23.72 | 79 | 0 | C5 | 71275 | rosids |
| 23.72 | 79 | 4 | C6 | 91835 | fabids |
| 19.52 | 65 | 0 | O | 3646 | Malpighiales |
| 19.52 | 65 | 0 | O1 | 3688 | Salicaceae |
| 19.52 | 65 | 0 | O2 | 238069 | Saliceae |
| 19.52 | 65 | 12 | G | 3689 | Populus |
| 14.11 | 47 | 47 | S | 3696 | Populus deltoides |
| 1.8 | 6 | 6 | S | 3693 | Populus tremuloides |
| 2.4 | 8 | 0 | O | 3744 | Rosales |
| 2.4 | 8 | 0 | O1 | 3487 | Moraceae |
| 2.4 | 8 | 0 | G | 66379 | Broussonetia |
| 2.4 | 8 | 8 | S | 172644 | Broussonetia papyrifera |
| 8.71 | 29 | 0 | C5 | 71274 | asterids |
| 8.41 | 28 | 0 | C6 | 91882 | campanulids |
| 8.41 | 28 | 0 | O | 4209 | Asterales |
| 8.41 | 28 | 0 | O1 | 4210 | Asteraceae |
| 8.11 | 27 | 0 | O2 | 102804 | Asteroideae |
| 7.81 | 26 | 0 | O3 | 102810 | Anthemideae |
| 7.81 | 26 | 0 | O4 | 886714 | Artemisiinae |
| 7.81 | 26 | 0 | G | 4219 | Artemisia |
| 7.81 | 26 | 26 | S | 55611 | Artemisia tridentata |
| 10.51 | 35 | 0 | C2 | 4447 | Liliopsida |
| 10.51 | 35 | 0 | C3 | 1437197 | Petrosaviidae |
| 10.51 | 35 | 0 | C4 | 4734 | commelinids |
| 10.51 | 35 | 0 | O | 38820 | Poales |
| 10.51 | 35 | 0 | O1 | 4479 | Poaceae |
| 8.71 | 29 | 0 | O2 | 359160 | BOP clade |
| 8.41 | 28 | 0 | O3 | 147368 | Pooideae |
| 8.41 | 28 | 0 | O4 | 1648037 | Poodae |
| 8.41 | 28 | 0 | O5 | 147387 | Poeae |
| 8.41 | 28 | 0 | O6 | 1652081 | Poeae Chloroplast Group 2 (Poeae type) |
| 8.41 | 28 | 0 | O7 | 640628 | Poinae |
| 8.41 | 28 | 0 | G | 4544 | Poa |
| 8.41 | 28 | 28 | S | 4545 | Poa pratensis |
| 1.8 | 6 | 0 | O2 | 147370 | PACMAD clade |
| 1.8 | 6 | 0 | O3 | 147369 | Panicoideae |
| 1.5 | 5 | 0 | O4 | 1648033 | Andropogonodae |
| 1.5 | 5 | 0 | O5 | 147429 | Andropogoneae |
| 1.5 | 5 | 0 | O6 | 1648029 | Tripsacinae |
| 1.5 | 5 | 0 | G | 4575 | Zea |
| 1.5 | 5 | 5 | S | 4577 | Zea mays |

**Negative control 2**

| Percentage of reads within taxon | Number of reads within taxon | Number of reads identified as taxon | Taxonomic rank | NCBI taxon ID | Taxon |
| --- | --- | --- | --- | --- | --- |
| 89.24 | 4505 | 4505 | U | 0 | unclassified |
| 10.76 | 543 | 0 | R | 1 | root |
| 10.76 | 543 | 0 | R1 | 131567 | cellular organisms |
| 10.76 | 543 | 0 | D | 2759 | Eukaryota |
| 10.76 | 543 | 3 | K | 33090 | Viridiplantae |
| 10.6 | 535 | 0 | P | 35493 | Streptophyta |
| 10.6 | 535 | 0 | P1 | 131221 | Streptophytina |
| 10.6 | 535 | 0 | P2 | 3193 | Embryophyta |
| 10.6 | 535 | 0 | P3 | 58023 | Tracheophyta |
| 10.6 | 535 | 0 | P4 | 78536 | Euphyllophyta |
| 10.6 | 535 | 0 | P5 | 58024 | Spermatophyta |
| 10.6 | 535 | 1 | C | 3398 | Magnoliopsida |
| 10.56 | 533 | 4 | C1 | 1437183 | Mesangiospermae |
| 7.67 | 387 | 0 | C2 | 71240 | eudicotyledons |
| 7.67 | 387 | 0 | C3 | 91827 | Gunneridae |
| 7.67 | 387 | 5 | C4 | 1437201 | Pentapetalae |
| 6.26 | 316 | 0 | C5 | 71275 | rosids |
| 6.06 | 306 | 4 | C6 | 91835 | fabids |
| 3.45 | 174 | 0 | O | 3744 | Rosales |
| 3.13 | 158 | 0 | O1 | 3487 | Moraceae |
| 3.13 | 158 | 0 | G | 66379 | Broussonetia |
| 3.13 | 158 | 158 | S | 172644 | Broussonetia papyrifera |
| 2.42 | 122 | 0 | O | 3646 | Malpighiales |
| 2.42 | 122 | 0 | O1 | 3688 | Salicaceae |
| 2.42 | 122 | 0 | O2 | 238069 | Saliceae |
| 2.42 | 122 | 2 | G | 3689 | Populus |
| 2.02 | 102 | 102 | S | 3696 | Populus deltoides |
| 2.75 | 139 | 1 | O1 | 4479 | Poaceae |
| 1.7 | 86 | 3 | O2 | 359160 | BOP clade |
| 1.58 | 80 | 0 | O3 | 147368 | Pooideae |
| 1.49 | 75 | 0 | O4 | 1648037 | Poodae |
| 1.49 | 75 | 0 | O5 | 147387 | Poeae |
| 1.49 | 75 | 0 | O6 | 1652081 | Poeae Chloroplast Group 2 (Poeae type) |
| 1.49 | 75 | 0 | O7 | 640628 | Poinae |
| 1.49 | 75 | 0 | G | 4544 | Poa |
| 1.49 | 75 | 75 | S | 4545 | Poa pratensis |
| 1.03 | 52 | 0 | O2 | 147370 | PACMAD clade |
| 1.03 | 52 | 0 | O3 | 147369 | Panicoideae |
